# Supplementary material for: Effects of psychosocial support interventions on survival in inpatient and outpatient healthcare settings: A meta-analysis of 106 randomized controlled trials
Source: PLoS Med. 2021 May 18;18(5):e1003595. doi: 10.1371/journal.pmed.1003595 (PMC8130925; doi:10.1371/journal.pmed.1003595)
Supplement: S1 Alternative Language Abstract — (PDF) [file pmed.1003595.s002.pdf]

Título

Efectos de las Intervenciones de Apoyo Psicosocial en la Supervivencia de Pacientes Hospitalizados y Ambulatorios: Un Metaanálisis de 106 Ensayos Controlados Aleatorios

Smith TB, Workman C, Andrews C, Barton B, Cook M, Layton R, Morrey A, Petersen D, Holt-Lunstad J. *PLOS Medicine;* 2021.

Resumen

Antecedentes

Los hospitales, clínicas y organizaciones de la salud han proporcionado intervenciones de apoyo psicosocial a pacientes para complementar sus tratamientos médicos. Revisiones de estudios previos sobre intervenciones que aumentan apoyo psicosocial en entornos médicos han reportado resultados variados. Este metaanálisis aborda las preguntas de cuán efectivas son las intervenciones de apoyo psicosocial en mejorar la supervivencia del paciente y qué características potenciales moderadoras están asociadas con una mayor efectividad.

Métodos y hallazgos

Se evaluaron los ensayos controlados aleatorios (ECA) de las intervenciones de apoyo psicosocial en pacientes hospitalizados y ambulatorios reportando datos de supervivencia, incluyendo estudios que reportan la mortalidad relacionada con la enfermedad o la mortalidad por todas otras causas. Las búsquedas bibliográficas incluyeron reportes de estudios desde enero de 1980 hasta octubre de 2020 de las bases de datos Embase, Medline, Cochrane Library, CINAHL, Alt Health Watch, PsycINFO, Social Work Abstracts y Google Scholar. Al menos dos evaluadores examinaron los estudios, recopilaron los datos y evaluaron su calidad. Como mínimo dos evaluadores independientes también recopilaron datos y evaluaron la calidad del estudio. Los datos de razón de probabilidades (odds ratio, OR) y razón de tasas (hazard ratio, HR) se analizaron por separado utilizando modelos de efectos aleatorios. De 42.054 estudios, 106 ECA que incluyeron 40.280 pacientes cumplieron criterios de inclusión. La edad promedio de los pacientes era de 57.2 años, con 52% de mujeres y 48% de hombres. El 42% tenía una enfermedad cardiovascular, el 36% cáncer y el 22% otras condiciones médicas. En 87 ECA que reportan datos para períodos de tiempo discretos, el promedio OR fue de 1,20 (IC del 95% = 1,09 a 1,31, $p <0,001$) indicando un aumento del 20% en la probabilidad de supervivencia entre los pacientes recibiendo apoyo psicosocial comparado con los que solo reciben tratamiento médico estándar. Entre esos estudios, las intervenciones psicosociales que explícitamente promueven comportamientos de salud produjeron una mayor probabilidad de supervivencia, mientras que intervenciones sin ese enfoque primario no lo hicieron. En 22 ECA que reportaron tiempo de supervivencia, el promedio del HR fue de 1,29 (IC del 95% = 1,12 a 1,49, $p <0,001$) indicando un aumento de 29% en la probabilidad de supervivencia entre pacientes que reciben la intervención y el grupo control. Entre esos estudios, las metarregresiones identificaron tres variables moderadoras: tipo de grupo de control, gravedad de la enfermedad del paciente y sesgos en la investigación. Los estudios en los que los grupos de control recibieron clases sobre salud además del tratamiento médico promediaron efectos más leves que aquellos en los que los grupos de control recibieron solo tratamiento médico. Los estudios con pacientes que tenían una gravedad de enfermedad relativamente mayor tendieron a obtener un incremento menor en el tiempo de supervivencia en comparación con los grupos de control. En uno de los tres análisis, los estudios con mayor riesgo de sesgos en la investigación tendieron a reportar mejores resultados. La limitación principal de los datos es que las intervenciones con poca frecuencia mantuvieron al personal y los participantes desinformados en cuanto tratamientos, de modo que no se controló las expectativas que pacientes tenían para mejorar.

Conclusiones

En este metaanálisis, los datos de OR indicaron que las intervenciones de apoyo psicosocial que promueven la motivación del paciente en realizar comportamientos de salud mejoraron la supervivencia del paciente, pero las intervenciones centradas principalmente en resultados sociales o emocionales de los pacientes no prolongaron la vida. Los datos de HR indicaron que las intervenciones psicosociales, predominantemente enfocadas en resultados sociales o emocionales, mejoraron la supervivencia, pero produjeron efectos similares al grupo con clases de salud y fueron menos efectivas entre los pacientes con una gravedad aparentemente mayor de la enfermedad. La posibilidad de sesgos en las investigaciones puede impactar la interpretación de los datos.

(Translation from English to Spanish by Laura Melgarejo Perez and Juan Valladares)

Reference

Smith, T. B., Workman, C., Andrews, C., Barton, B., Cook, M., Layton, R., Morrey, A., Petersen, D., & Holt-Lunstad, J. (2021). Effects of Psychosocial Support Interventions on Survival in Inpatient and Outpatient Health Care Settings: A Meta-Analysis of 106 Randomised Controlled Trials, *PLOS Medicine. DOI: 10.1371/journal.pmed.1003595*
