# Supplementary material for: Effects of psychosocial support interventions on survival in inpatient and outpatient healthcare settings: A meta-analysis of 106 randomized controlled trials
Source: PLoS Med. 2021 May 18;18(5):e1003595. doi: 10.1371/journal.pmed.1003595 (PMC8130925; doi:10.1371/journal.pmed.1003595)
Supplement: S2 Alternative Language Abstract — (PDF) [file pmed.1003595.s003.pdf]

標題 社會心理支持治療對住院患者的存活和門診醫療環境的影響：106 個隨機對照試驗的整合分析

Smith TB, Workman C, Andrews C, Barton B, Cook M, Layton R, Morrey A, Petersen D, Holt-Lunstad J. *PLOS Medicine;* 2021.

概要

背景

醫院、診所和衞生組織已為病患提供社會心理支持治療（psychosocial support intervention）以增補治癒性治療（curative treatment）。對於擴大在醫療環境中的社會心理支持，之前的分析匯報了參差不齊的結果。這個整合分析（meta-analysis）針對以下方面進行探討：（1）社會心理支持治療在改善患者存活方面的效果；以及（2）哪些潛在的調節特徵（moderators）與更高的療效相關。

方法和發現

我們評估了相關的隨機對照試驗（RCTs）中住院和門診醫療環境所報告的存活數據。當中包括疾病相關的或全因死亡率。文獻檢索包含由1980年1月至2020年10月期間的研究。我們在Embase, Medline, Cochrane Library, CINAHL, Alt Health Watch, PsycINFO, Social Work Abstracts, 和Google Scholar 數據庫進行檢索。最少由兩名評審員來篩檢研究，析取數據並評估研究質量，並由兩名獨立的評審員來篩檢研究和析取數據。我們用隨機效應模式（random effects model）來分別分析了比值比（odds ratio，OR）和風險比值（hazard ratio，HR）的數據。在42054項研究中，106項RCTs（包含了40280例患者）符合納入條件。患者平均年齡為57.2歲，其中女性為52%，男性為48%；42%的人患有心血管疾病，36%的人患有癌症，22%的人患有其他疾病。87個RCTs報告了離散時間段（discrete time periods）數據，平均值為OR＝1.20 （95% 信賴區間 ＝1.09 – 1.31， $p < 0.001$）。接受社會心理支持治療患者存活的可能性比接受常規治療的對照組提升了20%。社會心理支持治療明顯促進了健康的行為，從而提高存活的可能性。相反，沒有側重社會心理支持的治療方式並沒有這些成效。22個RCTs報告提供了存活時間（survival time）的數據，其平均值為HR＝1.29 （95% 信賴區間 ＝1.12 – 1.49， $p < 0.001$）。隨著時間的推移，接受社會心理支持治療的患者與對照組相比，存活概率增加了29%。整合迴歸（meta-regression）發現了三個調節變項（moderators）：對照組類型、患者疾病嚴重程度和研究偏差的風險。對照組中除常規治療外（treatment as usual，TAU），還接受健康教育的情況下，其平均效果要比僅接受TAU的對照組低。對疾病嚴重程度較高患者的數據顯示，他們在存活時間上，比對照組獲得相對較小的效益。其中一個分析顯示，存在較高研究偏差風險的研究傾向報告較好的結果。研究數據中最主要的限制是受試者與施測人員雙方在多數情況下知道受試者屬於實驗組或是對照組，因此沒有控制受試者對治療效果的預期的這個變數。

結論

OR數據顯示社會心理支持治療能促進患者的動力和自發地進行有利於健康的行為，從而提高患者的存活率。但是，主要針對患者的社交或情感方面結果的治療並不能延長其壽命

。HR數據顯示社會心理支持治療（主要針對社交或情感方面的結果）可改善存活率，但其產生的效果與接受健康教育相似，並在病情嚴重程度較高的患者中效果較差。存在研究偏差的風險可能影響到數據的解讀。

(Translation from English to Chinese Traditional Characters by Cheng Wai Man)

Reference

Smith, T. B., Workman, C., Andrews, C., Barton, B., Cook, M., Layton, R., Morrey, A., Petersen, D., & Holt-Lunstad, J. (2021). Effects of Psychosocial Support Interventions on Survival in Inpatient and Outpatient Health Care Settings: A Meta-Analysis of 106 Randomised Controlled Trials, *PLOS Medicine.* DOI: 10.1371/journal.pmed.1003595
