# Supplementary material for: Effects of psychosocial support interventions on survival in inpatient and outpatient healthcare settings: A meta-analysis of 106 randomized controlled trials
Source: PLoS Med. 2021 May 18;18(5):e1003595. doi: 10.1371/journal.pmed.1003595 (PMC8130925; doi:10.1371/journal.pmed.1003595)
Supplement: S3 Alternative Language Abstract — (PDF) [file pmed.1003595.s004.pdf]

标题 社会心理支持治疗对住院患者的存活和门诊治疗环境的影响：106 个随机对照试验的整合分析

Smith TB, Workman C, Andrews C, Barton B, Cook M, Layton R, Morrey A, Petersen D, Holt-Lunstad J. *PLOS Medicine;* 2021.

概要

背景

医院、诊所和卫生组织已为病患提供社会心理支持治疗（psychosocial support intervention）以增加治愈性治疗（curative treatment）。对于扩大在医疗环境中的社会心理支持，现存的分析报告中结果不一。这个整合分析（meta-analysis）针对以下方面进行探讨：（1）社会心理支持治疗在改善患者存活方面的效果；以及（2）与较高疗效关联的调节变量特征。

方法和发现

我们评估了随机对照试验（RCTs）中住院和门诊治疗环境所报告的关于存活的数据。这其中包含了对与疾病相关的或全因死亡率的研究。文献检索选取了来自 Embase, Medline, Cochrane Library, CINAHL, Alt Health Watch, PsycINFO, Social Work Abstracts, 和 Google Scholar 数据库中 1980 年 1 月至 2020 年 10 月期间的研究。我们至少采用两名评审员来筛检研究，选取数据并评估研究质量，并由两名独立的评审员来筛检研究和选取数据。我们用随机效应模式（random effects model）来分别分析了比值比（odd ratio，OR）和风险比值（hazard ratio, HR）的数据。在 42054 项研究中，106 项 RCTs（包含了 40280 例患者）符合入选标准。患者的平均年龄为 57.2 岁，其中女性占 52%，男性占 48%; 42%患有心血管疾病，36%患有癌症，22%患有其他疾病。87 个 RCTs 报告了离散时间段（discrete time periods）数据，平均值为 OR=1.20（95%置信区间 ＝1.09 – 1.31， p < 0.001）。接受社会心理支持治疗患者存活的可能性比接受常规治疗的对照组提升了 20%。社会心理治疗明显促进健康的行为，从而提高存活的可能性，反之，没有侧重社会心理支持的治疗方式则没有这些成效。22 个 RCTs 报告提供了存活时间（survival time）的数据，平均值为 HR＝1.209（95% 置信区间＝1.12 – 1.49， p < 0.001）。随着时间的推移，接受社会心理支持治疗的患者与对照组相比，存活概率增加了 29%。整合回归（meta-regression）一共发现了三个调节变量（moderators）：对照组类型，患者疾病严重程度，和研究偏差（research bias）的风险。对照组中除常规治疗（treatment as usual，TAU）外还接受健康教育的情况下，其平均效果要比仅接受 TAU 的对照组低。对疾病严重程度较高患者的研究显示，他们在存活时间上，比对照组获得了相对较小的收益。三项分析之一表明，存在较高研究偏差风险的研究倾向报告较好的结果。研究数据中最主要的限制是，受试者与施测人员双方在多数情况下知道受试者属于实验组或对照组，因此没有控制受试者对治疗效果的预期。

结论

OR 数据显示社会心理治疗能促进患者的动力和应对，从而参与健康的行为并提高患者的生存率。但是，主要针对患者的社交或情感方面结果的治疗并不能延长其寿命。HR 数据显示主要针对社交或情感方面的结果的社会心理支持治疗可改善存活率，但其所产生的效果与接受健康教育相似，并在病情严重程度较高的患者中效果较差。研究偏差的风险可能威胁到了数据的解读。

(Translation from English to Chinese Simplified Characters by Li Zhen)

Reference

Smith, T. B., Workman, C., Andrews, C., Barton, B., Cook, M., Layton, R., Morrey, A., Petersen, D., & Holt-Lunstad, J. (2021). Effects of Psychosocial Support Interventions on Survival in Inpatient and Outpatient Health Care Settings: A Meta-Analysis of 106 Randomised Controlled Trials, *PLOS Medicine.* DOI: 10.1371/journal.pmed.1003595
