# Supplementary material for: Effects of psychosocial support interventions on survival in inpatient and outpatient healthcare settings: A meta-analysis of 106 randomized controlled trials
Source: PLoS Med. 2021 May 18;18(5):e1003595. doi: 10.1371/journal.pmed.1003595 (PMC8130925; doi:10.1371/journal.pmed.1003595)
Supplement: S4 Alternative Language Abstract — (PDF) [file pmed.1003595.s005.pdf]

தலைப்பு:
உள்நோயாளி மற்றும் வெளிநோயாளர் ஆரோக்கியத்தில் உயிர்வாழ்வதற்கான உளவியல் சமூக ஆதரவு தலையீடுகளின் விளைவுகள் பராமரிப்பு அமைப்புகள்: 106 சீரற்ற கட்டுப்பாட்டு ஒரு மெட்டா (Meta)பகுப்பாய்வு.

Smith TB, Workman C, Andrews C, Barton B, Cook M, Layton R, Morrey A, Petersen D, Holt-Lunstad J. PLOS Medicine; 2021.

சுருக்கம் பின்னணி
மருத்துவமனைகள், கிளினிக்குகள் மற்றும் சுகாதார நிறுவனங்கள் மருத்துவ நோயாளிகளுக்கு மருத்துவ பராமரிப்புக்கு துணை மனநல தலையீடுகளை வழங்கியுள்ளன. மருத்துவ அமைப்புகளில் மனோசமூக ஆதரவை அதிகரிக்கும் தலையீடுகளின் முந்தைய மதிப்புரைகள் கலவையான விளைவுகளை அறிவித்தன. இந்த மெட்டா பகுப்பாய்வு நோயாளியின் உயிர்வாழ்வை மேம்படுத்துவதில் உளவியல் சமூக ஆதரவு தலையீடுகள் எவ்வளவு பயனுள்ளதாக இருக்கும் மற்றும் எந்த சாத்தியமான மிதமான அம்சங்கள் அதிக செயல்திறனுடன் தொடர்புடையவை என்ற கேள்விகளைக் குறிக்கிறது.

முறைகள் மற்றும் கண்டுபிடிப்புகள்

உள்நோயாளிகள் மற்றும் வெளிநோயாளிகளின் சுகாதாரப் பாதுகாப்பு அமைப்புகளில் உளவியல் ரீதியான ஆதரவு தலையீடுகளின் சீரற்ற கட்டுப்பாட்டு சோதனைகளை (ஆர்.சி.டி) மதிப்பீடு செய்துள்ளோம், உயிர்வாழும் தரவைப் புகாரளிக்கிறது, இதில் நோய் தொடர்பான அல்லது அனைத்து காரணங்களுக்கும் ஏற்படும் இறப்புகளைப் புகாரளிக்கும் ஆய்வுகள் அடங்கும். இலக்கியத் தேடல்களில் ஜனவரி, 1980 முதல் அக்டோபர், 2020 வரை எம்பேஸ், மெட்லைன், கோக்ரேன் நூலகம், CINAHL, Alt Health Watch, PsycINFO, Social Work Abstracts மற்றும் Google Scholar தரவுத்தளங்களிலிருந்து அணுகப்பட்ட ஆய்வுகள் அடங்கும். குறைந்தது இரண்டு விமர்சகர்கள் ஆய்வுகள், பிரித்தெடுக்கப்பட்ட தரவு மற்றும் மதிப்பிடப்பட்ட ஆய்வுத் தரத்தை திரையிட்டனர், குறைந்தது இரண்டு சுயாதீன விமர்சகர்கள் தரவைப்

பிரித்தெடுத்து ஆய்வு தரத்தை மதிப்பிடுகின்றனர். சீரற்ற விளைவு மாதிரிகள் பயன்படுத்தி ஒற்றை விகிதம் (OR, odds ratio) மற்றும் ஆபத்து விகிதம் (HR, hazard ratio) தரவு தனித்தனியாக பகுப்பாய்வு செய்யப்பட்டன. தேடப்பட்ட 42054 ஆய்வுகளில், 40280 நோயாளிகள் உட்பட 106 ஆர்.சி.டி.க்கள் சேர்க்கும் அளவுகோல்களை பூர்த்தி செய்தன. நோயாளியின் சராசரி வயது 57.2 ஆண்டுகள், 52% பெண்கள் மற்றும் 48% ஆண்கள்; 42% இருதய நோய், 36% புற்றுநோய், மற்றும் 22% பிற நோய்கள். தனித்துவமான கால அவகாசங்களுக்கான தரவைப் புகாரளிக்கும் 87 RCT களில், சராசரி OR = 1.20 (95% CI = 1.09 முதல் 1.31, ப <0.001) ஆகும், இது நிலையான மருத்துவத்தைப் பெறும் கட்டுப்பாட்டுக் குழுக்களுடன் ஒப்பிடும்போது உளவியல் ரீதியான ஆதரவைப் பெறும் நோயாளிகளிடையே உயிர்வாழ 20% அதிகரித்திருப்பதைக் குறிக்கிறது. பராமரிப்பு. அந்த ஆய்வுகளில், சுகாதார நடத்தைகளை வெளிப்படையாக ஊக்குவிக்கும் உளவியல் சமூக தலையீடுகள் உயிர்வாழ்வதற்கான மேம்பட்ட வாய்ப்பைக் கொடுத்தன, அதேசமயம் அந்த முதன்மை கவனம் இல்லாமல் தலையீடுகள் செய்யவில்லை. உயிர்வாழும் நேரத்தைப் புகாரளிக்கும் 22 RCT களில், சராசரி HR = 1.29 (95% CI = 1.12 முதல் 1.49 வரை, ப <0.001) ஆகும், இது கட்டுப்பாடுகளுடன் ஒப்பிடும்போது தலையீடு பெறுநர்களிடையே காலப்போக்கில் உயிர்வாழ்வதற்கான 29% அதிகரித்த நிகழ்தகவைக் குறிக்கிறது. அந்த ஆய்வுகளில், மெட்டா-பின்னடைவுகள் மூன்று மிதமான மாறிகள் அடையாளம் காணப்பட்டன: கட்டுப்பாட்டு குழு வகை, நோயாளி நோய் தீவிரம் மற்றும் ஆராய்ச்சி சார்பு ஆபத்து. கட்டுப்பாட்டு குழுக்கள் மருத்துவ சிகிச்சையை மட்டுமே பெற்றதை விட கட்டுப்பாட்டு குழுக்கள் மருத்துவ சிகிச்சையுடன் கூடுதலாக சுகாதார வகுப்புகளைப் பெற்ற ஆய்வுகள் பலவீனமான விளைவுகளைக் கொண்டிருந்தன. நோயாளிகளுடன் ஒப்பீட்டளவில் அதிக நோய் தீவிரம் கொண்ட ஆய்வுகள் கட்டுப்பாட்டு குழுக்களுடன் ஒப்பிடும்போது உயிர்வாழும் நேரத்தில் சிறிய லாபங்களைக் கொடுக்கின்றன. மூன்று பகுப்பாய்வுகளில் ஒன்றில், ஆராய்ச்சி சார்புக்கு அதிக ஆபத்து உள்ள ஆய்வுகள் சிறந்த முடிவுகளைப் புகாரளிக்க முனைகின்றன. தரவுகளின் முக்கிய வரம்பு என்னவென்றால், தலையீடுகள் எப்போதாவது பணியாளர்கள் மற்றும் பங்கேற்பாளர்களுக்கு சிகிச்சைகள் பற்றி தெரியாமல் வைத்திருக்கின்றன, அதாவது முன்னேற்றத்திற்கான நோயாளியின் எதிர்பார்ப்புகள் கட்டுப்படுத்தப்படவில்லை.

முடிவுரை

இந்த மெட்டா பகுப்பாய்வில், முரண்பாடுகளின் விகித தரவு, நோயாளிகளின் உந்துதலை ஊக்குவிக்கும் / சுகாதார நடத்தைகளில் ஈடுபடுவதை சமாளிக்கும் மனநல சமூக நடத்தை ஆதரவு தலையீடுகள் நோயாளியின் உயிர்வாழ்வை மேம்படுத்துவதாக சுட்டிக்காட்டின, ஆனால் நோயாளிகளின் சமூக அல்லது உணர்ச்சி விளைவுகளை மையமாகக் கொண்ட தலையீடுகள் ஆயுளை நீடிக்கவில்லை. ஆபத்து விகித தரவு, சமூக அல்லது உணர்ச்சி விளைவுகளில் முக்கியமாக கவனம் செலுத்தியது, உயிர்வாழ்வை மேம்படுத்தியது, ஆனால் சுகாதார வகுப்புகளுக்கு ஒத்த விளைவுகளை அளித்தது மற்றும் வெளிப்படையாக அதிக நோய் தீவிரத்தன்மை கொண்ட நோயாளிகளிடையே குறைவான செயல்திறனைக் கொண்டிருந்தது என்பதைக் குறிக்கிறது. ஆராய்ச்சி சார்பின் ஆபத்து என்பது தரவு விளக்கத்திற்கு ஒரு நம்பத்தகுந்த அச்சுறுத்தலாகும் .

(Translation from English to Tamil by Babu Manuel Abel)

Reference

Smith, T. B., Workman, C., Andrews, C., Barton, B., Cook, M., Layton, R., Morrey, A., Petersen, D., & Holt-Lunstad, J. (2021). Effects of Psychosocial Support Interventions on Survival in Inpatient and Outpatient Health Care Settings: A Meta-Analysis of 106 Randomised Controlled Trials, *PLOS Medicine.* DOI: 10.1371/journal.pmed.1003595
