# Supplementary material for: Effects of psychosocial support interventions on survival in inpatient and outpatient healthcare settings: A meta-analysis of 106 randomized controlled trials
Source: PLoS Med. 2021 May 18;18(5):e1003595. doi: 10.1371/journal.pmed.1003595 (PMC8130925; doi:10.1371/journal.pmed.1003595)
Supplement: S5 Alternative Language Abstract — (PDF) [file pmed.1003595.s006.pdf]

Titolo

Effetti di Interventi di Supporto Psicosociale sulla Sopravvivenza in Strutture Sanitarie Ambulatoriali e di Ricovero: Una Meta-Analisi di 106 Studi Randomizzati Controllati.

Smith TB, Workman C, Andrews C, Barton B, Cook M, Layton R, Morrey A, Petersen D, Holt-Lunstad J.
PLOS Medicine; 2021.

Astratto

Contesto di Sfondo

Ospedali, cliniche ed organizzazioni sanitarie hanno fornito interventi di supporto psicosociale a pazienti medici per integrare le cure mediche.

Le revisioni precedenti degli interventi che aumentano il supporto psicosociale in contesti medici hanno riportato risultati contrastanti.

Questa meta-analisi si domanda quanto siano efficaci gli interventi di supporto psicosociale nel migliorare la sopravvivenza del paziente e quali potenziali caratteristiche sono associate ad una maggiore efficacia.

Metodi e Risultati

Abbiamo valutato studi randomizzati controllati (SRC) di interventi di supporto psicosociale in strutture sanitarie ambulatoriali e di ricovero riportando dati di sopravvivenza, includendo studi che riportano mortalità correlata alla malattia o per tutte le altre cause.

Ricerche della letteratura hanno incluso studi riportati dal Gennaio 1980 all'Ottobre 2020 accessibili tramite i database Embase, Medline, Cochrane Library, CINAHL, Alt Health Watch, PsycINFO, Social Work Abstracts e Google Scholar. Almeno due revisori hanno analizzato gli studi, estratto i dati e valutato la qualità degli studi, con almeno due revisori indipendenti che hanno anche estratto i dati e valutato la qualità degli studi.

I dati sul rapporto di probabilità (odds ratio, OR) e sull'azzardo relativo (hazard ratio, HR) sono stati analizzati separatamente utilizzando modelli con effetti casuali.

Dei 42054 studi cercati, 106 SRC comprendenti 40280 pazienti hanno soddisfatto i criteri di inclusione. L'età media dei pazienti era di 57,2 anni, con il 52% di sesso femminile e il 48% di sesso maschile; il 42% aveva malattie cardiovascolari, il 36% aveva il cancro e il 22% aveva altre condizioni.

Negli 87 SRC che hanno riportato dati per periodi di tempo discreti, la media è stata OR = 1,20 (95% IC = 1,09-1,31, p < 0,001), indicando un aumento del 20% della probabilità di sopravvivenza tra i pazienti che ricevono supporto psicosociale rispetto ai gruppi di controllo che ricevono solo trattamento medico. Tra questi studi, gli interventi psicosociali che promuovono esplicitamente comportamenti di salute hanno prodotto una maggiore probabilità di sopravvivenza, mentre gli interventi senza questo obiettivo primario non lo hanno fatto.

Nei 22 SRC che riportavano il tempo di sopravvivenza, la media era HR = 1,29 (95% CI = 1,12-1,49, p <0,001), indicando, nell'arco del tempo, un aumento del 29% nella probabilità di sopravvivenza tra i destinatari dell'intervento rispetto al gruppo di controllo.

Tra questi studi, le meta-regressioni hanno identificato tre variabili moderatrici: il tipo di gruppo di controllo, la gravità della malattia del paziente e il rischio di parzialità di ricerca.

Gli studi in cui i gruppi di controllo hanno ricevuto corsi sulla salute oltre al trattamento abituale (TA) hanno ottenuto in media effetti più deboli rispetto a quelli in cui i gruppi di controllo hanno ricevuto solo il TA.

Gli studi con pazienti con una gravità della malattia relativamente maggiore tendevano a produrre guadagni minori nel tempo di sopravvivenza rispetto ai gruppi di controllo. In una delle tre analisi, gli studi con un rischio più elevato di bias di ricerca tendevano a riportare risultati migliori.

La principale limitazione dei dati è che gli interventi hanno raramente impedito al personale ed ai partecipanti di essere consapevoli dei trattamenti, in modo tale che le aspettative di miglioramento non erano controllate.

Conclusioni

In questa meta-analisi, i dati di OR hanno indicato che gli interventi di supporto comportamentale psicosociale che promuovono la motivazione del paziente per impegnarsi in comportamenti salutari hanno migliorato la sopravvivenza del paziente, ma gli interventi incentrati principalmente sui risultati sociali o emotivi dei pazienti non hanno prolungato la vita.

I dati sulle HR hanno indicato che gli interventi psicosociali, prevalentemente incentrati sugli esiti sociali o emotivi, hanno migliorato la sopravvivenza, ma hanno prodotto

effetti simili a corsi sulla salute ed erano meno efficaci tra i pazienti con una gravità della malattia apparentemente maggiore. Il rischio di bias di ricerca è una minaccia plausibile per l'interpretazione dei dati.

(Translation from English to Italian by Claudia Mencarelli and Tommaso Cardullo)

Reference

Smith, T. B., Workman, C., Andrews, C., Barton, B., Cook, M., Layton, R., Morrey, A., Petersen, D., & Holt-Lunstad, J. (2021). Effects of Psychosocial Support Interventions on Survival in Inpatient and Outpatient Health Care Settings: A Meta-Analysis of 106 Randomised Controlled Trials, *PLOS Medicine.* DOI: 10.1371/journal.pmed.1003595
