# Supplementary material for: Effects of psychosocial support interventions on survival in inpatient and outpatient healthcare settings: A meta-analysis of 106 randomized controlled trials
Source: PLoS Med. 2021 May 18;18(5):e1003595. doi: 10.1371/journal.pmed.1003595 (PMC8130925; doi:10.1371/journal.pmed.1003595)
Supplement: S6 Alternative Language Abstract — (PDF) [file pmed.1003595.s007.pdf]

Başlık

Psikososyal Destek Müdahalelerinin Yatan ve Ayakta Sağlık Hizmet Veren Merkezlerde Sağkalım Üzerine Etkileri: 106 Randomize Kontrollü Çalışmanın Meta Analizi

Smith TB, Workman C, Andrews C, Barton B, Cook M, Layton R, Morrey A, Petersen D, Holt-Lunstad J.
PLOS Medicine; 2021.

Özet
Arkaplan

Hastaneler, klinikler ve sağlık kuruluşları, tedavi edici bakımı desteklemek için tıbbi hastalara psikososyal destek müdahaleleri sağlamıştır. Tıbbi ortamlarda psikososyal desteği artıran müdahaleler hakkında daha önceden yapılan incelemeler karışık sonuçlar bildirmiştir. Bu meta-analiz, psikososyal destek müdahalelerinin hasta sağkalımını iyileştirmede ne kadar etkili olduğunu ve hangi potansiyel düzenleyici özelliklerinin daha etkili olduğu ile ilgili soruları ele almaktadır.

Yöntemler ve bulgular

Hastalıkla ilgili veya tüm nedenlere bağlı ölüm oranlarını bildiren çalışmalar da dahil olmak üzere, hayatta kalma verilerini bildiren yatan ve ayakta tedavi veren merkezlerde psikososyal destek müdahaleleri ile ilişkili randomize kontrollü çalışmalar (RKÇ) değerlendirilmiştir. Literatür taramaları, Embase, Medline, Cochrane Library, CINAHL, Alt Health Watch, PsycINFO, Social Work Abstracts ve Google Scholar veritabanlarından erişilen ve Ocak 1980'den Ekim 2020'ye kadar bildirilen çalışmaları kapsamaktadır. En az iki hakem, tüm çalışmaları taradı, verileri çıkardı ve çalışma kalitesini değerlendirdi; en az iki bağımsız hakem de verileri çıkardı ve çalışma kalitesini değerlendirdi. Olasılık oranı (OR, odds ratio) ve tehlike oranı (TO, hazard ratio) verileri, rastgele etki ağırlıklı modeller kullanılarak ayrı ayrı analiz edildi. Taranan 42054 çalışmadan 40280 hastayı içeren 106 RKÇ dahil edilme kriterlerini karşıladı. Hastaların ortalama yaşı 57,2 olup, % 52'si kadın ve % 48'i erkek; % 42'sinde kardiyovasküler hastalık, % 36'sında kanser ve % 22'sinde diğer başka hastalıklar olduğu tespit edildi. Farklı zaman dilimleri içinde veri bildiren 87 RKÇ genelinde ortalama OR = 1,20 olarak saptandı (% 95 CI = 1,09 ila 1,31, p <0,001) ve standart tıbbi destek alan kontrol gruplarına kıyasla psikososyal destek alan hastalar arasında hayatta kalma olasılığının% 20 arttığını gösterildi. Bu çalışmalar arasında, sağlık davranışlarını açıkça destekleyen psikososyal müdahaleler hayatta kalma olasılığını artırırken, bu birincil odağı olmayan müdahalelerde fark gözlenmedi. Sağkalım süresini bildiren 22 RKÇ'de ortalama TO = 1.29 (% 95 CI = 1.12 ila 1,49, p <0,001) olup, kontrollere kıyasla müdahale uygulananlar arasında zaman içinde % 29 artış saptanan hayatta kalma olasılığını göstermektedir. Bu çalışmalar arasında, meta-regresyonlar üç düzenleyici değişken tanımladı: Kontrol grubu tipi, hastanın hastalık ciddiyeti ve araştırma taraflılığı riski. Kontrol gruplarının normal şekilde tedaviye (NŞT) ek olarak sağlık bilgisi/dersleri aldığı çalışmalar, kontrol gruplarının sadece NŞT aldığı çalışmalara göre daha zayıf etkileri gözlendi. Nispeten daha yüksek hastalık ciddiyetine sahip hastalarla yapılan çalışmalar, kontrol gruplarına göre hayatta kalma süresinde daha küçük kazanımlar sağlama eğilimindeydi. Üç analizden birinde, araştırma taraflılığı riski daha yüksek olan çalışmalar daha iyi sonuçlar bildirme eğilimindeydi. Verilerin temel kısıtlaması, müdahalelerin çok

nadiren personelde ve katılımcılarda çalışma gruplarına karşı çift kör yürütülebilmesidir, bu nedenle iyileştirme beklentileri kontrol edilememiştir.

Sonuçlar

Bu meta-analizde, OR verileri sağlık davranışlarında hasta motivasyonunu/başa çıkmasını teşvik eden psikososyal davranışsal destek müdahalelerinin hasta sağkalımını iyileştirdiğini, ancak öncelikle hastaların sosyal veya duygusal sonuçlarına odaklanan müdahalelerin yaşamı uzatmadığını gösterdi. TO verileri, ağırlıklı olarak sosyal veya duygusal sonuçlara odaklanan psikososyal müdahalelerin sağkalımı iyileştirdiğini, ancak sağlık bilgisine/derslerine benzer etkiler sağladığını ve görünüşte daha yüksek hastalık şiddetine sahip hastalar arasında daha az etkili olduğunu gösterdi. Araştırma taraflılığı riski, verilerin yorumlanması için kabul edilebilir bir risk olmaya devam etmektedir.

(Translation from English to Turkish by Murat Çakır)

Reference

Smith, T. B., Workman, C., Andrews, C., Barton, B., Cook, M., Layton, R., Morrey, A., Petersen, D., & Holt-Lunstad, J. (2021). Effects of Psychosocial Support Interventions on Survival in Inpatient and Outpatient Health Care Settings: A Meta-Analysis of 106 Randomised Controlled Trials, *PLOS Medicine.* DOI: 10.1371/journal.pmed.1003595
