# Supplementary material for: Effects of psychosocial support interventions on survival in inpatient and outpatient healthcare settings: A meta-analysis of 106 randomized controlled trials
Source: PLoS Med. 2021 May 18;18(5):e1003595. doi: 10.1371/journal.pmed.1003595 (PMC8130925; doi:10.1371/journal.pmed.1003595)
Supplement: S7 Alternative Language Abstract — (PDF) [file pmed.1003595.s008.pdf]

Titel
Effekte von psychosozialen Unterstützungsmaßnahmen auf das Überleben in stationären und ambulanten Gesundheitseinrichtungen: Eine Meta-Analyse von 106 randomisierten, kontrollierten Studien
Smith TB, Workman C, Andrews C, Barton B, Cook M, Layton R, Morrey A, Petersen D, Holt-Lunstad J. *PLOS Medicine*; 2021.

Abstrakt

Hintergrund

Krankenhäuser, Kliniken und Gesundheitsorganisationen haben medizinischen Patienten psychosoziale Unterstützungsmaßnahmen zur Verfügung gestellt, um die medizinische Versorgung zu ergänzen. Frühere Übersichten über Maßnahmen zur Ergänzung der psychosozialen Unterstützung in medizinischen Einrichtungen haben über gemischte Ergebnisse berichtet. Diese Meta-Analyse befasst sich mit der Frage, wie effektiv psychosoziale Unterstützungimaßnahmen das Überleben von Patienten verbessern und welche potenziell moderierenden Merkmale mit größerer Effektivität verbunden sind.

Methoden und Ergebnisse

Wir bewerteten randomisierte kontrollierte Studien (RCTs) zu psychosozialen Unterstützungsmaßnahmen in stationären und ambulanten Gesundheitseinrichtungen, die Überlebensdaten berichteten, einschließlich Studien, die krankheitsbezogene oder Gesamtsterblickeit berichteten. Die Literatursuche umfasste Studien, die von Januar 1980 bis Oktober 2020 in den Datenbanken Embase, Medline, Cochrane Library, CINAHL, Alt Health Watch, PsycINFO, Social Work Abstracts und Google Scholar veröffentlicht wurden. Mindestens zwei Reviewer sichteten die Studien, extrahierten die Daten und bewerteten die Studienqualität, wobei mindestens zwei unabhängige Reviewer ebenfalls die Daten extrahierten und die Studienqualität bewerteten. Chancenverhältnis (*OR*, odds ratio) und Risikoquotient (*HR,* hazard ratio) Daten wurden separat mit Random Effects-Modellen analysiert. Von 42054 recherchierten Studien erfüllten 106 RCTs mit 40280 Patienten die Aufnahmekriterien. Das Durchschnittsalter der Patienten betrug 57,2 Jahre, 52 % waren weiblich und 48 % männlich; 42 % hatten eine kardiovaskuläre Erkrankung, 36 % eine Krebserkrankung und 22 % hatten andere Erkrankungen. Über 87 RCTs hinweg, die Daten für diskrete Zeiträume berichteten, betrug der Durchschnitt *OR* = 1,20 (95% CI = 1,09 bis 1,31, *p* < 0,001), was auf eine um 20% erhöhte Überlebenswahrscheinlichkeit bei Patienten hinweist, die psychosoziale Unterstützung erhielten, verglichen mit Kontrollgruppen, die eine medizinische Standardversorgung erhielten. In diesen Studien führte psychosoziale Unterstützung, die explizit das Gesundheitsverhalten förderte, zu einer verbesserten Überlebenswahrscheinlichkeit, während Unterstützung ohne diesen primären Fokus dies nicht taten. In 22 RCTs, die die Überlebenszeit berichteten, betrug der Durchschnitt *HR* = 1,29 (95% CI = 1,12 bis 1,49, *p* < 0,001), was auf eine um 29% erhöhte Überlebenswahrscheinlichkeit auf Zeit bei den Unterstützungsempfängern im Vergleich zu den Kontrollgruppen hinweist. Bei diesen Studien identifizierten Regressionsanalyse drei moderierende Variablen: den Typ der Kontrollgruppe, den Schweregrad der Erkrankung der Patienten und das Risiko eines Forschungsbias. Studien, in denen die Kontrollgruppen zusätzlich zur medizinischen Behandlung Gesundheitskurse erhielten, zeigten im Durchschnitt schwächere Wirkung als die, in denen die Kontrollgruppen nur medizinische Behandlung erhielten. Studien mit Patienten, die einen relativ höheren Krankheitsschweregrad hatten, ergaben tendenziell eine geringere Zunahme in Überlebenszeit im Vergleich zu den Kontrollgruppen. In einer von drei Analysen berichteten Studien mit einem höheren Risiko für Forschungsbias tendenziell bessere Ergebnisse. Die größte Einschränkung der Daten ist, dass das Personal und die Teilnehmer bei den Unterstützungsmaßnahmen oft über die

Behandlungen informiert wurden, so dass die Erwartungen der Patienten auf eine Verbesserung nicht kontrolliert wurden.

Schlussfolgerungen

In dieser Meta-Analyse wiesen Chancenverhältnisdaten darauf hin, dass psychosoziale Verhaltensunterstützungsmaßnahmen, die die Motivation/den Umgang der Patienten mit Gesundheitsverhalten fördern, das Überleben der Patienten verbesserten, aber Maßnahmen, die sich primär auf die sozialen oder emotionalen Ergebnisse der Patienten konzentrieren, das Leben nicht verlängerten. Risikoquotientdaten (HR) wiesen darauf hin, dass psychosoziale Maßnahmen, die sich vorwiegend auf soziale oder emotionale Ergebnisse konzentrierten, das Überleben verbesserten, aber ähnliche Effekte wie Gesundheitsklassen erzielten und bei Patienten mit offensichtlich höherem Krankheitsschweregrad weniger wirksam waren. Das Risiko eines Forschungsbias ist eine plausible Gefahr für die Interpretation der Daten.

(Translation from English to German by Samira Herber)

Reference

Smith, T. B., Workman, C., Andrews, C., Barton, B., Cook, M., Layton, R., Morrey, A., Petersen, D., & Holt-Lunstad, J. (2021). Effects of Psychosocial Support Interventions on Survival in Inpatient and Outpatient Health Care Settings: A Meta-Analysis of 106 Randomised Controlled Trials, *PLOS Medicine.* DOI: 10.1371/journal.pmed.1003595
