# Supplementary material for: Effects of psychosocial support interventions on survival in inpatient and outpatient healthcare settings: A meta-analysis of 106 randomized controlled trials
Source: PLoS Med. 2021 May 18;18(5):e1003595. doi: 10.1371/journal.pmed.1003595 (PMC8130925; doi:10.1371/journal.pmed.1003595)
Supplement: S8 Alternative Language Abstract — (PDF) [file pmed.1003595.s009.pdf]

Judul

Pengaruh Intervensi Dukungan Psikososial terhadap Kelangsungan Hidup di Area Layanan Kesehatan Rawat Inap dan Rawat Jalan: Sebuah Meta-analisis dari 106 Uji Acak Terkendali

Smith TB, Workman C, Andrews C, Barton B, Cook M, Layton R, Morrey A, Petersen D, Holt-Lunstad J.

PLOS Medicine; 2021.

Abstrak

Latar Belakang

Rumah sakit, klinik, dan organisasi kesehatan telah menyediakan intervensi dukungan psikososial kepada para pasien untuk melengkapi layanan kesehatan. Tinjauan-tinjauan sebelumnya terhadap intervensi dalam menambah dukungan psikososial di area kesehatan menunjukkan hasil yang beragam. Meta-analisis ini membahas beberapa pertanyaan tentang seberapa efektif intervensi dukungan psikososial dalam meningkatkan kelangsungan hidup pasien dan aspek khusus potensial apa saja yang terkait dengan tingkat efektivitas yang lebih baik.

Metode dan Temuan

Kami mengevaluasi uji acak terkendali (RCT) dari intervensi dukungan psikososial di area layanan kesehatan rawat inap dan rawat jalan yang melaporkan data kelangsungan hidup, termasuk laporan studi yang berkaitan dengan penyakit atau semua penyebab kematian. Penelusuran literatur mencakup studi yang dilaporkan sejak Januari 1980 hingga Oktober 2020 dan diakses dari *Embase, Medline, Cochrane Library, CINAHL, Alt Health Watch, PsycINFO*, Abstrak Pekerjaan Sosial serta pangkalan data Google Cendekia. Setidaknya terdapat dua penelaah yang bertugas dalam meninjau studi, mengekstraksi data, dan menilai kualitas studi. Disamping itu, setidaknya dua peninjau independen lainnya juga mengekstraksi data dan menilai kualitas studi. Data *odds* rasio (OR) dan rasio bahaya (HR) dianalisis secara terpisah menggunakan model efek acak. Dari hasil penelusuran 4.2054 studi, 106 RCT termasuk 40.280 pasien memenuhi kriteria inklusi. Usia rata-rata pasien adalah 57,2 tahun, dengan 52% perempuan dan 48% laki-laki; 42% menderita penyakit kardiovaskular, 36% menderita kanker, dan 22% memiliki kondisi lain. Dari 87 data pelaporan RCT untuk periode waktu tertentu, rata-rata OR = 1,20 (95% CI = 1,09 sampai 1,31, p <0,001). Hal ini menunjukkan peningkatan kemungkinan bertahan hidup sebanyak 20% di antara pasien yang menerima dukungan psikososial dibandingkan dengan kelompok-kelompok kontrol yang menerima layanan kesehatan standar. Di antara studi tersebut, intervensi psikososial secara eksplisit mendukung perilaku kesehatan yang menghasilkan peluang bertahan hidup menjadi lebih baik, sedangkan intervensi tanpa fokus utama tersebut tidak menunjukkan hasil yang demikian. Pelaporan waktu bertahan hidup pada 22 RCT menunjukkan rata-rata HR = 1,29 (95% CI = 1,12 sampai 1,49, p <0,001), dimana peluang bertahan hidup dari waktu ke waktu meningkat sebanyak 29% di antara penerima intervensi dibandingkan dengan kontrol. Di antara studi tersebut, meta-regresi mengidentifikasi tiga komponen: tipe kelompok kontrol, keparahan penyakit pasien, dan risiko bias penelitian. Studi dimana kelompok-kelompok kontrol menerima kelas kesehatan selain perawatan medis menunjukkan efek rerata lebih lemah dibandingkan dengan kelompok kontrol yang hanya menerima perawatan medis. Studi dengan pasien-pasien yang memiliki tingkat keparahan penyakit relatif lebih tinggi cenderung menunjukkan kemajuan yang lebih rendah dalam waktu bertahan hidup relatif dibandingkan dengan kelompok-kelompok kontrol. Dalam salah satu dari tiga analisis, studi-studi dengan risiko bias penelitian yang lebih tinggi cenderung menunjukkan hasil yang lebih baik. Keterbatasan utama dari data adalah bahwa intervensi-intervensi jarang membuat para peserta dan personel mendapatkan informasi mengenai perawatan, sehingga harapan pasien untuk kondisi yang lebih baik tidak terpantau.

Kesimpulan

Dalam meta-analisis ini, data *odds* rasio menunjukkan bahwa intervensi dukungan perilaku psikososial yang mempromosikan motivasi pasien / mampu terlibat dalam perilaku kesehatan dapat meningkatkan kelangsungan hidup pasien, akan tetapi intervensi yang hanya fokus pada hasil sosial atau emosional pasien tidak memperpanjang hidup. Data rasio bahaya mengindikasikan bahwa intervensi psikososial yang fokus utamanya pada hasil sosial atau emosional mampu meningkatkan kelangsungan hidup, namun menghasilkan efek yang sama terhadap kelas kesehatan dan kurang efektif di antara pasien-pasien dengan tingkat keparahan penyakit yang tampak lebih tinggi. Risiko bias penelitian adalah kemungkinan pengaruh pada interpretasi data.

(Translation from English to Indonesian by Pungki Lupiyaningdyah)

Reference

Smith, T. B., Workman, C., Andrews, C., Barton, B., Cook, M., Layton, R., Morrey, A., Petersen, D., & Holt-Lunstad, J. (2021). Effects of Psychosocial Support Interventions on Survival in Inpatient and Outpatient Health Care Settings: A Meta-Analysis of 106 Randomised Controlled Trials. *PLOS Medicine.* DOI: 10.1371/journal.pmed.1003595
