# Supplementary material for: Effects of psychosocial support interventions on survival in inpatient and outpatient healthcare settings: A meta-analysis of 106 randomized controlled trials
Source: PLoS Med. 2021 May 18;18(5):e1003595. doi: 10.1371/journal.pmed.1003595 (PMC8130925; doi:10.1371/journal.pmed.1003595)
Supplement: S9 Alternative Language Abstract — (PDF) [file pmed.1003595.s010.pdf]

العنوان:

آثار ادخال الدعم النفسي والاجتماعي على فرص البقاء على قيد الحياة عند المرضى الداخلين وغير الداخلين في محيط الرعاية الصحية: تحليل وصفي لمئة وست تجارب عشوائية محكمة.

الملخص

تمهيد

المستشفيات, العيادات والمنظمات الصحية تقدم للمرضى دعم نفسي- اجتماعي وذلك لدعم الرعاية الطبية. ملخصات الدراسات والابحاث المنشورة السابقة لهذا التدخل لرفع الدعم النفسي والاجتماعي في المحيط الطبي انتجت مخرجات متباينة. هذا التحليل الوصفي يعالج التساؤلات حول مدى فعالية التدخل عن طريق الدعم النفسي- اجتماعي في تحسين نسب بقاء المريض على قيد الحياة و ما هي الميزات المعتدلة المحتملة التي ترافق تحقيق فعالية اكبر.

الاسلوب البحثي والنتائج

لقد قمنا بتقييم تجارب محكمة عشوائية لادخال الدعم النفسي- اجتماعي لمحيط البيئة الصحية للمرضى الداخلين وغير الداخلين لاظهار معلومات النجاة وبما في ذلك دراسات تشير الى نسب الوفيات المرتبطة بمرض ونسب الوفيات لجميع الاسباب. الأبحاث السابقة تضمنت دراسات للفترة من كانون الثاني لعام 1980 ولغاية تشرين اول لعام 2020 تم التوصل اليها من خلال,Embase, Medline, CINAHL, ALT Health Watch, PsycINFO, مكتبة Cochrane ,ملخصات العمل الاجتماعي, قاعدة بيانات الباحثين في غوغل.

هناك باحثان مراجِعان اثنان على الاقل تفحصا الدراسات,واستخلصا البيانات وقاما بتقييم جودة الدراسة وذلك بالاضافة الى باحثين مراجعين مستقلين على الاقل لاستخلاص البيانات وتقييم جودة الدراسة. بيانات نسبة الارجحية ونسبة المخاطرة تم تحليلها بشكل مستقل باستخدام عامل التأثير العشوائي. ما بين 42054 دراسة تم بحثها, هناك 106 تجارب عشوائية محكمة تضمنت 40280 مريض طابقت معيار الدمج. المعدل العمري للمرضى كان 57.2 سنة, مع 52% إناث و 48% ذكور, 42% منهم مصابين بامراض الأوعية الدموية, و36% منهم مصابين بالسرطان, و22% مصابين بحالات اخرى. ما بين 87 تقرير للحالات العشوائية المحكمة لفترات زمنية منفصلة, معدل نسبة الارجحية كان يساوي 1.20 (95% CI = 1.09 to 1.31, p < 0.001) والتي تشير الى ان نسبة الزيادة في احتمالية النجاة بين المرضى الذين تلقوا الدعم النفسي والاجتماعي كانت 20% مقارنة بمجموعات محكمة تلقت الرعاية الطبية المعتادة. من ضمن تلك الدراسات, تدخل الدعم النفسي والاجتماعي دعم بشكل واضح السلوك الصحي نتج عنه تحسن احتمالية النجاة, في حين التدخلات الاخرى بدون التركيز الاساسي على مثل هذا التدخل لم ينتج عنها مثل هذا التحسن. من بين 22 حالة محكمة عشوائية بينت معدل النجاة عبر الوقت, معدل نسبة المخاطرة كان 1.29 (95% CI = 1.12 to 1.49, p < 0.001) =والذي يشير الى زيادة احتمالية النجاة خلال الزمن بنسبة 29% بين متلقي الدعم مقارنة بغيرهم.

من خلال هذه الدراسات, العلاقة الوصفية اعتمدت على ثلاثة متغيرات معتدلة: نوع المجموعة التي لم تتلق التدخل, شدة مرض المريض واحتمالية التحيز البحثي. الدراسات على المجموعات المحكمة التي تلقت حصص صحية بالإضافة الى العلاج الطبي كان معدل التأثيرات فيها اضعف من المجموعات التي تلقت العلاج الطبي فقط. الدراسات على المرضى المصابين بمرض اشد حدة نسبيا اظهرت نتائج اقل في مدة النجاة مقارنة بالمجموعات المحكمة. في واحد من التحليلات الثلاثة, الدراسات مع نسبة مخاطرة اكبر بالتحيز البحثي اظهرت نتائج افضل. المحدودية الرئيسية في البيانات هي ان التدخلات بشكل غير متكرر جعلت من طاقم العمل والمرضى غير مطلعين على العلاجات, وعليه فان توقعات المرضى للتحسن لم يمكن التحكم بها.

الخاتمة

في هذا التحليل الوصفي, بيانات نسبة الارجحية اظهرت ان دعم السلوك النفسي والاجتماعي لتعزيز حافز المريض للاندماج في السلوك الصحي حسن من فرص المريض للبقاء على قيد الحياة, غير ان التدخلات التي تركز بشكل اساسي على مخرجات المريض الاجتماعية والعاطفية لم تطل في حياتهم. بيانات نسبة المخاطرة اظهرت ان التدخلات النفسية والاجتماعية التي ركزت في الغالب الاعم على النتائج الاجتماعية او العاطفية حسنت من فرص البقاء على قيد الحياة لكنها اظهرت آثار مشابهة للصفوف الصحية وكانت اقل تأثيرا بين المرضى الذي تظهر عليهم شدة المرض بشكل اكبر. خطورة التحيز البحثي تشكل تهديد معقول لتفسير البيانات.

(Translation from English to Arabic by Sara Abu Al-Samen)

Reference

Smith, T. B., Workman, C., Andrews, C., Barton, B., Cook, M., Layton, R., Morrey, A., Petersen, D., & Holt-Lunstad, J. (2021). Effects of Psychosocial Support Interventions on Survival in Inpatient and Outpatient Health Care Settings: A Meta-Analysis of 106 Randomised Controlled Trials, *PLOS Medicine.* DOI: 10.1371/journal.pmed.1003595
