# Supplementary material for: Effects of psychosocial support interventions on survival in inpatient and outpatient healthcare settings: A meta-analysis of 106 randomized controlled trials
Source: PLoS Med. 2021 May 18;18(5):e1003595. doi: 10.1371/journal.pmed.1003595 (PMC8130925; doi:10.1371/journal.pmed.1003595)
Supplement: S10 Alternative Language Abstract — (PDF) [file pmed.1003595.s011.pdf]

Título

Efeitos das intervenções de apoio psicossocial na sobrevivência em ambientes de cuidados de saúde em regime de internamento e ambulatório: Uma meta-análise de 106 ensaios controlados randomizados.

Smith TB, Workman C, Andrews C, Barton B, Cook M, Layton R, Morrey A, Petersen D, Holt-Lunstad J. *PLOS Medicine;* 2021.

Resumo

Fundo contextual

Hospitais, clínicas e organizações de saúde forneceram aos pacientes médicos intervenções de apoio psicossocial para complementar os cuidados médicos. Avaliações anteriores de intervenções que aumentam o apoio psicossocial em ambientes médicos relataram resultados variados. Esta meta-análise aborda as questões de quão eficazes são as intervenções de apoio psicossocial na melhoria da sobrevida do paciente e quais recursos moderadores potenciais estão associados a uma maior eficácia.

Métodos e descobertas

Avaliamos ensaios controlados randomizados (ECR) de intervenções de apoio psicossocial em ambientes hospitalares e ambulatoriais relatando dados de sobrevivência, incluindo estudos relatando mortalidade relacionada à doença ou por todas as causas. Pesquisas de literatura incluíram estudos relatados de janeiro de 1980 a outubro de 2020 acessados em Embase, Medline, Cochrane Library, CINAHL, Alt Health Watch, PsycINFO, Social Work Abstracts e bancos de dados Google Scholar. Pelo menos dois revisores examinaram os estudos, extraíram os dados e avaliaram a qualidade do estudo, com pelo menos dois revisores independentes também extraindo dados e avaliando a qualidade do estudo. Os dados da razão de chances (odds ratio, OR) e da razão de risco (hazard ratio, HR) foram analisados separadamente usando modelos de efeitos aleatórios. Dos 42054 estudos, 106 ECRs incluindo 40280 pacientes preencheram os critérios de inclusão. A idade média dos pacientes foi de 57,2 anos, sendo 52% mulheres e 48% homens; 42% tinham doenças cardiovasculares, 36% tinham câncer e 22% tinham outras condições. Em 87 ECRs relatando dados para períodos de tempo distintos, a média foi OR = 1,20 (IC de 95% = 1,09 a 1,31, $p < 0,001$), indicando um aumento de 20% na probabilidade de sobrevivência entre os pacientes que recebem apoio psicossocial em comparação com grupos de controle que recebem cuidados médico padrão. Entre esses estudos, as intervenções psicossociais que promovem explicitamente os comportamentos de saúde aumentaram a probabilidade de sobrevivência, ao passo que as intervenções sem esse enfoque principal não o fizeram. Em 22 ECRs relatando tempo de sobrevivência, a média foi HR = 1,29 (IC 95% = 1,12 a 1,49, $p < 0,001$), indicando um aumento de 29% na probabilidade de sobrevivência ao longo do tempo entre os receptores de intervenção em comparação com os controles.

Entre esses estudos, as metarregressões identificaram três variáveis moderadoras: tipo de grupo de controle, gravidade da doença do paciente e risco de viés de pesquisa. Os estudos nos quais os grupos de controle receberam aulas de saúde além do tratamento médico apresentaram efeitos menores do que aqueles em que os grupos de controle receberam apenas tratamento médico. Estudos com pacientes com gravidade da doença relativamente maior tenderam a produzir ganhos menores no tempo de sobrevivência em relação aos grupos de controle. Em uma das três análises, os estudos com maior risco de viés de pesquisa tenderam a relatar melhores resultados. A principal limitação dos dados é que as intervenções raramente mantiveram o pessoal e os participantes desinformados sobre os tratamentos, de modo que as expectativas do paciente quanto à melhora não foram controladas.

Conclusões

Nesta meta-análise, os dados da razão de probabilidade (OR) indicaram que as intervenções de suporte psicossocial comportamental que promovem a motivação do paciente para se envolver em comportamentos de saúde, melhoraram a sobrevida do paciente, mas as intervenções com foco principalmente nos resultados sociais ou emocionais dos pacientes não prolongaram a vida. Os dados da razão de risco (HR) indicaram que as intervenções psicossociais, predominantemente focadas em resultados sociais ou emocionais, melhoraram a sobrevivência, mas produziram efeitos semelhantes às classes de saúde e foram menos eficazes entre os pacientes com gravidade de doença aparentemente maior. O risco de viés de pesquisa é uma ameaça plausível à interpretação dos dados.

(Translation from English to Portuguese by Solange Andrezzo and Larissa Vecchi)

Reference

Smith, T. B., Workman, C., Andrews, C., Barton, B., Cook, M., Layton, R., Morrey, A., Petersen, D., & Holt-Lunstad, J. (2021). Effects of Psychosocial Support Interventions on Survival in Inpatient and Outpatient Health Care Settings: A Meta-Analysis of 106 Randomised Controlled Trials, *PLOS Medicine.*
