# Supplementary material for: Effects of psychosocial support interventions on survival in inpatient and outpatient healthcare settings: A meta-analysis of 106 randomized controlled trials
Source: PLoS Med. 2021 May 18;18(5):e1003595. doi: 10.1371/journal.pmed.1003595 (PMC8130925; doi:10.1371/journal.pmed.1003595)
Supplement: S1 Text — (PDF) [file pmed.1003595.s012.pdf]

S1 Text. Literature search strategies and selection criteria.

Literature Searches

The searches included the following terms related to research design, psychosocial support interventions, and patient survival that were used for each of the databases listed below.

EMBASE

(“community based care”:ti,ab or “Community health worker-based intervention”:ti,ab or “coping skills intervention”:ti,ab or “coping skills training”:ti,ab or “couple-based intervention”:ti,ab or “diagnosis related group”:ti,ab or “diagnosis-related groups”/exp or “Family-Centered Empowerment Model”:ti,ab or “Family-led rehabilitation”:ti,ab or “group counseling”:ti,ab or “group education programme”:ti,ab or “group support”:ti,ab or “group therapies”:ti,ab or “group therapy”:ti,ab or “home-based support”:ti,ab or “Integrated care intervention”:ti,ab or “interpersonal counselling”:ti,ab or “Meaning-making intervention”:ti,ab or “meaning-centered”:ti,ab or “peer support”:ti,ab or “peer-support groups”:ti,ab or “peer-support intervention”:ti,ab or “psychol* interv*”:ti,ab or “psycholog* treat*”:ti,ab or “Psychological support”:ti,ab or “Psychosocial group”:ti,ab or “psychosocial group”:ti,ab or “psychosocial intervention”:ti,ab or “psychosocial intervention”:ti,ab or “psychosocial nursing intervention”:ti,ab or “psychosocial program”:ti,ab or “psychosocial program”:ti,ab or “psychosocial rehabilitation”:ti,ab or “psychosocial therapy”:ti,ab or “psychosocial therapy”:ti,ab or “psychosocial treatment”:ti,ab or “psychotherapeutic support”:ti,ab or “specialized home care intervention”:ti,ab or “reciprocal support”:ti,ab or “support group intervention”:ti,ab or “support intervention”:ti,ab or “support service”:ti,ab or “support* counsel*”:ti,ab or “supported self-management”:ti,ab or “supportive expressive group therapy”:ti,ab or “supportive-expressive group therapy”:ti,ab or “survivor group”:ti,ab or “social support”:ti,ab or “social engagement”:ti,ab or “social participation”:ti,ab or “interpersonal support”:ti,ab)
AND
('mortality'/exp OR 'survival'/exp OR 'longevity'/exp OR 'death'/exp OR 'loss of life' OR 'remain* living' OR 'fatality'/exp OR died OR 'remain* alive')
AND
('randomized controlled trial'/exp OR 'controlled trial, randomized' OR 'randomised control*' OR 'randomized control*' OR 'trial, randomized controlled' OR 'clinical trial'/exp OR ‘random* alloc*’ OR ‘random* assign*’ OR ‘assign* random*’ OR ‘alloc* random*’)
NOT
(palliative or hospice or (CBT and peptide) or dementia or Alzheimers or “infant mortality” or “infant death” or “fetus mortality” or “fetal mortality” or “fetal death” or “fetus survival” or “infant survival” or stillbirth or stillborn or (bereav* and (“death of a child” or “death of a spouse” or “loss of a child” or “loss of a spouse”)) or rats or mice or animal* or nonhuman)

Medline, CINAHL, Alt Health Watch, PsycINFO, and Social Work Abstracts

(“community based care” or “Community health worker-based intervention” or “coping skills intervention” or “coping skills training” or “couple-based intervention” or “diagnosis related group” or “diagnosis-related groups” or “Family-Centered Empowerment Model” or “Family-led rehabilitation” or “group counseling” or “group education programme” or “group support” or “group therapies” or “group therapy” or “home-based support” or “Integrated care intervention” or “interpersonal counselling” or “Meaning-making intervention” or “meaning-centered” or “peer support” or “peer-support groups” or “peer-support intervention” or “psychol* interv*” or “psycholog* treat*” or “Psychological support” or “Psychosocial group” or “psychosocial group” or “psychosocial intervention” or “psychosocial intervention” or “psychosocial nursing intervention” or “psychosocial program” or “psychosocial program” or “psychosocial rehabilitation” or “psychosocial therapy” or “psychosocial therapy” or “psychosocial

treatment” or “psychotherapeutic support” or “specialized home care intervention” or “reciprocal support” or “support group intervention” or “support intervention” or “support service” or “support* counsel*” or “supported self-management” or “supportive expressive group therapy” or “supportive-expressive group therapy” or “survivor group” or “social support” or “social engagement” or “social participation” or “interpersonal support”)
AND
('mortality' OR 'surviv*' OR 'longevity' OR 'death' OR 'loss of life' OR 'remain* living' OR 'fatality' OR 'fatal case' OR 'fatal outcome' OR died OR 'remain* alive’ or deceased or dead or dying)
AND
('randomized controlled trial'/exp OR 'controlled trial, randomized' OR 'randomised control*' OR 'randomized control*' OR 'trial, randomized controlled' OR 'clinical trial'/exp OR ‘random* alloc*’ OR ‘random* assign*’ OR ‘assign* random*’ OR ‘alloc* random*’)
NOT
(palliative or hospice or (CBT and peptide) or dementia or Alzheimers or “infant mortality” or “infant death” or “fetus mortality” or “fetal mortality” or “fetal death” or “fetus survival” or “infant survival” or stillbirth or stillborn or (bereav* and (“death of a child” or “death of a spouse” or “loss of a child” or “loss of a spouse”)) or rats or mice or animal* or nonhuman)

Google Scholar

Multiple synonyms were used, including:
"social support intervention" OR "group therapy" OR "psychotherapy group" OR “support group” OR "self-help groups" OR “support intervention” OR "emotional support" OR psychosocial OR coping
AND
mortality OR survival OR died
AND
"controlled trial" OR "cox regression" OR "clinical trial" OR randomized OR randomly

Cochrane Library

(Fatal Outcomes OR Mortality OR All Cause Mortality OR Survival OR Died In Hospital) AND (Death OR Cancer OR Cardiovascular OR Diabetes Mellitus OR Human Immunodeficiency Virus OR Stroke OR Kidney Disease OR Renal Dialysis OR Tumor Finding OR Carcinoma Of Lung OR Hospitalization OR Hospital Based Outpatient Care) AND (Social Support OR Psychosocial Intervention Strategy OR Psychosocial Care OR Psychosocial Procedure OR Group Psychotherapy OR Support Group Facilitation OR Emotional Support OR Supportive Verbal Psychotherapy)
AND
(“coping skills intervention” or “coping skills training” or “couple-based intervention” or “diagnosis related group” or “diagnosis-related groups” or “Family-Centered Empowerment Model” or “Family-led rehabilitation” or “group counseling” or “group education programme” or “group support” or “group therapies” or “group therapy” or “home-based support” or “Integrated care intervention” or “interpersonal counselling” or “Meaning-making intervention” or “meaning-centered” or “peer support” or “peer-support groups” or “peer-support intervention” or “psychological intervention” or “community based care” or “Community health worker-based intervention” or “psychological treatment” or “Psychological support” or “Psychosocial group” or “psychosocial group” or “psychosocial intervention” or “psychosocial intervention” or “psychosocial nursing intervention” or “psychosocial program” or “psychosocial program” or “psychosocial rehabilitation” or “psychosocial therapy” or “psychosocial therapy” or “psychosocial treatment” or “psychotherapeutic support” or “specialized home care intervention” or “reciprocal support” or “support group intervention” or “support intervention” or “support service” or “supportive counseling”

or “supported self-management” or “supportive expressive group therapy” or “supportive-expressive group therapy” or “survivor group” or “social support” or “social engagement” or “social participation” or “interpersonal support”)
AND
('mortality' OR 'survival' OR 'longevity' OR 'death' OR 'loss of life' OR 'remained living' OR 'fatality' OR 'fatal case' OR 'fatal outcome' OR died OR 'remained alive’ or deceased or dead or dying)
AND
('randomized controlled trial' OR 'controlled trial, randomized' OR 'randomised controlled' OR 'randomized control*' OR 'trial, randomized controlled' OR 'clinical trial'/exp OR ‘randomly allocated’ OR ‘randomly assigned’ OR ‘assigned randomly” OR ‘allocated randomly’)

Search terms specific to individual psychotherapy and disease management were removed based on journal reviewer feedback received on October, 2020 to refine inclusion criteria.

To reduce inadvertent omissions, we searched databases yielding the most citations (Embase, Medline, PsycINFO) more than once. We also manually examined the reference sections of past reviews and also of studies meeting the inclusion criteria to locate articles not identified in the database searches. Consistent with the PRISMA Statement, study selection occurred in two stages. Study titles and abstracts were independently screened by at least two reviewers. Full text of relevant studies were examined using inclusion/exclusion criteria. Reasons for exclusion were recorded and reported in Figure 1 in the manuscript.

In addition to database searches, we contacted authors whose publications had met inclusion criteria to request additional studies, published or unpublished.

Inclusion/Exclusion Prospective Criteria

The following PICO criteria were applied to published or unpublished reports in any language, which were translated to English or read/coded in the original language when team members were fluent in that language.

Participants/Population
Only medical patients with a disease associated with a probable cause of death were included. We excluded patients who only had dementia or mental illness but no other physical health condition. We excluded individuals recruited outside health care settings, including Alcoholics Anonymous and similar 12-step support groups. When multiple publications included the same study participants, we selected data from the longest follow-up or largest sample size if the follow-up length was equal across reports.

Interventions
Eligible interventions delivered real-time social, emotional, and/or psychological support. Such interventions could be in-person or using technology connecting people at a distance (e.g., telephone, computer, tablet). Such interventions could be provided one-on-one or in groups (and we coded for intervention format). Given that we were interested in the interventions' affect on longevity, we excluded interventions not intended to prolong life, including hospice and palliative care. We excluded interventions involving nonhuman support (e.g., higher power, pets).

Comparator(s)/control
This meta-analysis was restricted to randomised controlled trials. We included studies in which the comparison group either did not receive an intervention (e.g., treatment as usual or wait list control group) or received non-supportive attention, such as psychoeducational information about disease management (and we coded the differences). We excluded studies that only compared one bona fide psychosocial support to another.

Outcome
We extracted data regarding survival/mortality in terms of both (1) survival time (reported as hazard ratios) and (2) binary survival at a fixed timepoint (reported/calculated as odds ratios). We excluded data collapsing mortality with morbidity.

Inclusion/Exclusion Revised Criteria

Reviewer feedback (October, 2020) on our original manuscript submission to *PLOS Medicine* consistently indicated that our inclusion/exclusion criteria were too broad and required specification. In particular, concerns were raised about the inclusion of psychotherapy and disease management programs. We therefore re-screened studies to make certain that psychosocial support was a primary objective of the study, removing disease management programs (e.g., symptom monitoring, reminder messaging, psychoeducation) but retaining psychosocial interventions supportive of health behaviors. We also excluded studies providing solely one-on-one psychotherapy (because those provided a distinct kind of support, deserving of separate consideration). We included CBT or psychotherapy when it was one component of a larger intervention with other components that clearly met inclusion criteria (e.g., family support meetings). We retained group psychotherapy studies because that treatment modality primarily involves peer group interactions, albeit usually facilitated by professionals rather than medical staff; the kind of social/emotional support provided in a psychotherapy group strongly overlaps with what is typically provided in a support group not based on psychotherapy. To make all inclusion/exclusion criteria clearer to readers, we report the revised criteria in Table 1 in the body of the manuscript.
