# Supplementary material for: Effects of psychosocial support interventions on survival in inpatient and outpatient healthcare settings: A meta-analysis of 106 randomized controlled trials
Source: PLoS Med. 2021 May 18;18(5):e1003595. doi: 10.1371/journal.pmed.1003595 (PMC8130925; doi:10.1371/journal.pmed.1003595)
Supplement: S2 Text — (PDF) [file pmed.1003595.s013.pdf]

S2 Text. Data abstraction and analyses.

Data Abstraction

In our prospective planning, we sought to decrease the likelihood of human error in coding data, so a team of two raters coded each article. Then a different team of two raters independently coded the same article. This redundancy was intended to increase the accuracy of coding and data entry.

When multiple effect sizes were reported in a study at the same point in time (e.g., across different subsamples), we averaged the several values (weighted by SE) to avoid violating the assumption of independent samples. When a study contained multiple effect sizes across time, we extracted the data from the longest follow-up period (or larger sample size if follow-up length was equal across reports). We sought *OR* and *HR* data as effect sizes, but when studies reported other statistics (e.g., regression coefficients, Cohen's *d*, frequency counts), we transformed those values to *OR* using multiple online effect size calculators. We sought data from multivariable models, but we coded univariate data when that was the only option. When mortality was tracked over time but no participants died in either condition, we coded the effect size as OR = 1.

Data extraction involved:

• Information about the study: research design, type of control condition, year of study initiation, country;

• Participant characteristics of age, sex, medical diagnosis at intake, percentage of patients dying by endpoint, setting (inpatient, outpatient, or both);

• Information about the intervention type (e.g., one-on-one support, support group), focus (e.g., behavioral support, social/emotional support), delivery (e.g., in-person, telephone/online), number of sessions, length of sessions, duration of sessions in months, length of follow-up period after completion of intervention, and effectiveness of the intervention (e.g., statistically significant increases in patient perceptions of social support or decreases in patient distress);

• Effect sizes in terms of survival time (reported as hazards ratios) or binary survival at a fixed point in time (reported/calculated as odds ratios) and corresponding standard error.

• Risk of bias, as indicated by study-level reports of: random sequence generation, allocation concealment, blinding of participants and personnel, blinding of outcome assessment, incomplete participation data, selective reporting, balance of groups on variables at baseline, intention-to-treat analysis, and same conditions between intervention and control group, apart from the intervention itself. Ratings for each indicator were: low risk of bias, unclear risk of bias, and high risk of bias.

Extracted data were cross-checked to identify discrepancies. Discrepancies were resolved through discussion following scrutiny of the manuscript to the point of consensus or arbitration by the first or last author. Incomplete effect size data were requested via email from the study's corresponding author.

Strategy for Data Analyses

We prospectively planned to analyze and report: study, intervention, and participant characteristics; omnibus effect sizes across both *OR* and *HR* metrics; heterogeneity statistics; subgroup analyses and meta-regressions; and analyses of likelihood of publication bias. Omnibus, subgroup, and meta-regression analyses were conducted using random-effects weighted models calculated by STATA 16, SPSS 25, and Comprehensive Meta-Analysis version 3.0. To observe heterogeneity, we computed both *Q* and its level of statistical significance and the I^2 statistic and its 95% confidence interval. We did not pre-specify which variables to include in the meta-regressions, but we clustered them logically according to study, intervention, and participant characteristics. To address the possible influence of study rigor on the findings, we included study risk of bias as a predictor in all meta-regressions. We report the results of a subgroup analysis comparing behavioral support with social/emotional support based on comments of a reviewer to a prior version of this manuscript. We prospectively planned to evaluate the likelihood of publication bias estimates using the following methods: Funnel plots, the trim and fill method, and Egger's and Peters' regression tests.
