# Supplementary material for: Effects of psychosocial support interventions on survival in inpatient and outpatient healthcare settings: A meta-analysis of 106 randomized controlled trials
Source: PLoS Med. 2021 May 18;18(5):e1003595. doi: 10.1371/journal.pmed.1003595 (PMC8130925; doi:10.1371/journal.pmed.1003595)
Supplement: S1 Table — (PDF) [file pmed.1003595.s014.pdf]

S1 Table. PICOT inclusion criteria.

**P**opulation	We included studies of patients with a health condition likely to result in death if untreated, and who were recruited from health care settings (e.g., hospitals, clinics, inpatient/outpatient databases). We retained studies in which patients initially identified in health care settings received subsequent rehabilitation or home visits. We excluded patients with solely mental health disorders (e.g., anxiety, dementia) because other than increased likelihood of suicide, accidents, and possibly violence, those conditions only indirectly contribute to death if untreated. We retained studies in which patients had mental health conditions in addition to physical conditions resulting in death if untreated. We excluded patients receiving hospice or palliative care since we believed those interventions deserved separate review.
**I**ntervention	We included interventions explicitly intended to improve patient psychosocial functioning through social support, emotional coping, and/or distress reduction. As the majority of interventions described in the literature involve multiple components, we included interventions with mixed components (e.g., group meetings, nurse visits, telephone support) and coded for differences to compare outcomes.<br>We excluded studies not providing real-time interactions with humans and those providing only psychoeducation. We excluded interventions consisting solely of one-on-one psychotherapy, which historically has been a distinct kind of intervention (deserving separate systematic review), but we retained group psychotherapy interventions because the active social/emotional support provided is similar to support groups. We excluded interventions providing only disease management (e.g., patient symptom monitoring, case coordination) but included interventions including psychosocial support for health behaviors (e.g., motivation to engage in physical exercise, support for dietary adherence) for purposes of comparison. We excluded hospice or palliative care interventions since the observed outcome of this meta-analysis was survival among patients receiving curative or rehabilitative care.
**C**omparison	We included studies with control groups consisting of patients from the same area(s) who received curative or rehabilitative care for the same health condition(s) as the intervention group. We included control groups receiving health information/classes but excluded groups receiving another psychosocial intervention.
**O**utcome	We included studies reporting either fixed-time mortality or survival time. We excluded data combining mortality with morbidity/hospitalization.
**T**imeframe	We included only RCTs reported in the literature between January 1980 and October 2020.
