# Supplementary material for: Effects of psychosocial support interventions on survival in inpatient and outpatient healthcare settings: A meta-analysis of 106 randomized controlled trials
Source: PLoS Med. 2021 May 18;18(5):e1003595. doi: 10.1371/journal.pmed.1003595 (PMC8130925; doi:10.1371/journal.pmed.1003595)
Supplement: S2 Table — OR, odds ratio. (PDF) [file pmed.1003595.s015.pdf]

S2 Table. Characteristics of 87 psychosocial intervention studies reporting odds ratios of medical patient survival.

Study	Preexisting Condition	Intervention Format	Intervention Components	Avg. Age at Intake	Percent Died	Study Length (Months)	*N*	*OR*	95% CI
Albus et al (2009)	CVD	In-person group meetings Exercise, relaxation, group discussion	Supportive group discussions, behavioral-focused exercise, relaxation	54.2	7%	72.0	73	**1.59**	0.25, 10.13
Andryukhin et al (2010)	CVD	Individual & group meetings	Supportive consultations, coping, behavioral lifestyle education, exercise	67.0	4%	18.0	85	**2.21**	0.19, 25.28
Aranda et al (2006)	Cancer	Combination of formats	Empathetic support, coping and communication strategies	56.0	9%	3.0	69	**1.25**	0.31, 5.12
Armes et al (2007)	Cancer	In-person individual meetings	Behavioral support, emotional and behavioral coping	59.1	25%	9.0	60	**1.00**	0.31, 3.20
Arving et al (2007)	Cancer	Combination of formats	Problem solving, relaxation, and communication strategies	55.0	5%	24.0	179	**2.20**	0.53, 9.12
Badger et al (2012)	Cancer	Telephone meetings with family invited	Interpersonal psychotherapy, cancer education, and social support	47.3	1%	4.0	90	**0.33**	0.01, 8.22
Bambauer et al (2005)	CVD	Individual telephone meetings	Goal oriented, issue-focused and coping psychotherapy	60.2	0%	6.0	110	**3.45**	0.14, 86.79
Baucom et al (2009)	Cancer	In-person group meetings	Couples-based relationship communication and education	50.0	7%	12.0	14	**4.64**	0.16, 135.5
Beresnevaite et al (2000)	CVD	In-person group meetings	Supportive, progressive-relaxation, and emotional expression-based group therapy	50.2	11%	24.0	37	**1.20**	0.15, 9.57
Berger et al (2008)	HIV	In-person group meetings	Psychoeducation, group cohesion exercises, cognitive restructuring, and progressive muscle relaxation.	43.9	1%	12.0	71	**0.28**	0.01, 7.14
Björneklett et al (2013)	Cancer	In-person group meetings	Cancer information, coping strategies, and relaxation	58.3	14%	108.0	382	**0.67**	0.34, 1.31
Blumenthal et al (2005)	CVD	In-person group meetings	Stress management through exercise, coping, and social support	63.0	0%	48.0	86	**1.00**	0.02, 51.55
Blumenthal et al (2014)	COPD	Telephone meetings involving others	Stress management, coping, physical activity, supportive counseling	66.1	1%	52.8	326	**2.72**	0.71, 10.43
Blumenthal et al (2016)	CVD	In-person group meetings	Stress management training, education, group support, cognitive therapy, and exercise	61.0	3%	64.0	223	**6.71**	0.37, 120.8
Boesen et al (2011)	Cancer	In-person group meetings	Stress management, problem-solving, coping, and cognitive reframing	53.2	5%	48.0	205	**0.48**	0.12, 1.97
Burell (1994)	CVD	In-person group meetings	Group stress management and cognitive restructuring	56.2	8%	12.0	47	**4.18**	0.18, 97.86
Burell (1996)	CVD	In-person group meetings	Behavioral education, coping, and cognitive restructuring	58.0	9%	60.0	261	**2.36**	0.94, 5.95
Chan et al (2014)	T2D	Telephone meetings involving peers	Behavioral-focused peer support intervention	54.7	0%	12.0	628	**1.49**	0.25, 8.95
Claesson (2006)	CVD	In-person group meetings	Cognitive-behavioral stress management: education, self-	59.9	1%	36.0	197	**5.26**	0.25, 111.9

			monitoring, skills training, cognitive restructuring, social support						
Classen et al (2008)	Cancer	In-person group meetings	Build new social support structures through supportive-expressive group therapy	49.8	1%	24.0	357	**5.03**	0.24, 105.5
Cockcroft et al (1987)	CRD	Individual at home visits	Behavioral education and social support from assigned nurse	69.7	14%	10.0	73	**3.32**	0.78, 14.05
Colella (2009)	CVD	Telephone meetings involving peers	Emotional assistance, assigned peer social support	63.5	1%	3.0	209	**0.16**	0.01, 4.04
Creber et al (2016)	CVD	Individual meetings with mixed delivery	Behavioral, motivational interviewing and support from nurse	62.0	7%	3.0	100	**0.93**	0.17, 5.08
Cunningham et al (1998)	Cancer	In-person group meetings	Support group; coping skills, group psychosocial therapy	50.5	77%	60.0	66	**1.13**	0.33, 3.84
Cuong et al (2016)	HIV	Individual at home visits	Peer-support and adherence counseling	31.9	11%	24.0	640	**1.02**	0.62, 1.68
Edelman et al (1999)	Cancer	In-person group meetings	Group CBT, expressing emotions and building group support	49.7	91%	60.0	92	**0.95**	0.60, 1.50
Evans et al (1995)	Cancer	In-person group meetings	Cognitive behavioral group sessions focused on reducing anxiety and depression	54.0	37%	60.0	51	**1.02**	0.33, 3.18
Fawzy et al (2003)	Cancer	In-person group meetings	Stress management, relaxation, coping, behavioral education, and social support	42.5	29%	120.0	68	**1.33**	0.47, 3.79
Foley et al (2010)	Cancer	In-person group meetings	Mindfulness, meditation and group discussion	55.5	4%	3.0	115	**0.29**	0.03, 2.91
Fors (2018)	COPD or CVD	Telephone meetings	Person-centered communication skills and goals	77.6	15%	6.0	243	**1.34**	0.66, 2.71
Frasure-Smith et al (1997)	CVD	In-person individual meetings	Emotional support, reassurance, and practical medical advice	59.3	5%	12.0	1376	**0.71**	0.43, 1.17
Friedman et al (1986)	CVD	In-person group meetings	Group type-A behavioral and cardiac counseling	53.3	6%	54.0	771	**1.41**	0.76, 2.64
Frizelle et al (2004)	CVD	In-person individual and group meetings	Behavioral exercise and education-based intervention with focus on group support	61.5	5%	3.0	22	**3.95**	0.14, 108.1
Guo et al (2013)	Cancer	In-person group meetings	Psychoeducation, cognitive-behavioral therapy, and supportive-expressive therapy	47.0	16%	24.0	178	**1.08**	0.50, 2.35
Hanssen et al (2009)	CVD	Telephone meetings	Stress-management, coping, and education individualized to patient	60.1	6%	18.0	288	**0.88**	0.30, 2.61
Härter et al (2016)	All outpatient	Telephone meetings	Behavioral motivational interviewing and support	69.4	5%	24.0	6125	**1.77**	1.38. 2.27
Hawkes et al (2012)	CVD	Telephone meetings	Support and encouragement for self-management, health coaching, emotional well-being	60.6	0%	6.0	430	**0.20**	0.01, 4.15

Heisler et al (2013)	CVD	Combination of formats	Peer partner assigned support coupled with goal-centered group sessions	69.1	12%	12.0	266	**0.84**	0.40, 1.75
Herrmann-Lingen et al (2016)	CVD	In-person individual and group meetings	Supportive-expressive one-on-one and group psychotherapy	59.2	3%	24.0	570	**1.52**	0.53, 4.32
Hjelle et al (2019)	Stroke	In-person individual meetings	Establishing relationship with nurse, coping, and problem solving	65.5	0%	6.0	322	**3.21**	0.13, 79.4
Holtmaat et al (2019)	Cancer	In-person group meetings	Meaning-centered supportive group psychotherapy	58.0	2%	26.0	170	**0.27**	0.01, 5.41
Horlick et al (1984)	CVD	In-person group meetings with spouse	Behavioral education via group discussion, involving spouse	53.5	7%	6.0	101	**0.37**	0.04, 3.24
Hossain et al (2017)	Spinal Cord Injury	Combination of formats	Behavioral-focused advice and support for family and individual	31.5	7%	24.0	30	**1.00**	0.06, 17.62
Høybye et al (2010)	Cancer	Online group meetings	Internet-based peer support groups	54.0	4%	13.0	789	**0.89**	0.43, 1.82
Hynninen et al (2010)	COPD	In-person group meetings	Group CBT	60.9	2%	8.0	51	**5.20**	0.24, 114.0
Ibfelt et al (2011)	Cancer	In-person group meetings	Psychosocial residential rehabilitation intervention including lectures and peer group discussions	60.5	2%	6.0	452	**5.22**	0.62, 43.70
Irvine et al (2010)	CVD	Combination of formats	Counseling and psychoeducational; booklet including enhancing social network, love and intimacy	65.6	4%	12.0	193	**3.10**	0.61, 15.75
Johansen et al (2003)	CVD	In-person group meetings with spouse	Group based psychosocial rehabilitation	63.0	22%	60.0	166	**1.65**	0.78, 3.48
Johansson et al (2016)	T2D	In-person group meetings	Peer support, group meetings, exercise	63.0	3%	24.0	305	**0.66**	0.17, 2.51
Jones et al (1996)	CVD	In-person individual and group meetings	Group psychological therapy, counseling, relaxation, stress management	65.0	12%	12.0	2314	**1.08**	0.78, 1.48
Koertge et al (2008)	CVD	In-person group meetings	Stress management: including interpersonal conflict	62.1	3%	24.0	247	**6.59**	0.80, 54.40
Lamers et al (2010)	T2D or COPD	Individual at-home visits	CBT and self-management; reattribution of negative cognitions and problem solving	70.7	2%	9.0	314	**0.76**	0.15, 3.83
Lee et al (2006)	Cancer	In-person individual meetings	Meaning-making intervention: emotional and cognitive support	56.7	1%	NR	82	**0.33**	0.01, 8.22
Lewin et al (2009)	CVD	Combination of formats	Relaxation, emotional coping, cognitive behavioral rehabilitation, family involvement	61.1	3%	6.0	192	**1.17**	0.21, 6.55
Liljeroos et al (2015)	CVD	In-person individual meetings	Psychoeducational support in patient-partner dyads	71.3	25%	24.0	134	**1.52**	0.68, 3.42
Lindley et al (2017)	Stroke	In-person family meetings	Family-led rehabilitation	57.7	12%	6.0	1250	**1.22**	0.87, 1.70
Liu et al (2018)	Stroke	In-person individual meetings	Family-centered recovery and self-care ability training	56.7	7%	6.0	128	**9.00**	1.09, 74.22

May et al (2009)	Cancer	In-person individual and group meetings	Physical training and CBT, physical activity, household, hobbies, family relationships, and social contacts	48.8	4%	12.0	147	**1.63**	0.26, 10.07
Mayou et al (2002)	CVD	Combination of formats	Individualized information; emotional, cognitive, behavioral advice; partner involved	58.1	3%	12.0	114	**1.47**	0.24, 9.16
McArdle et al (1996)	Cancer	Combination of formats	Psychosocial support: information, counselling, and regular group meetings	56.7	5%	12.0	152	**0.75**	0.17, 3.26
McKinley et al (2009)	CVD	Combination of formats	Informational, emotional, and social support	67.2	2%	12.0	3522	**1.24**	0.75, 2.06
Meneses et al (2007)	Cancer	Combination of formats	Psychoeducational support group	54.5	0%	6.0	261	**2.95**	0.12, 73.19
Minet et al (2011)	T1D or T2D	In-person individual meetings	Supportive group education program	56.5	0%	24.0	349	**2.95**	0.12, 72.89
Nakimuli-Mpungu et al (2015)	HIV	In-person group meetings	Group support psychotherapy	44.5	3%	8.0	109	**0.54**	0.05, 6.13
Nakimuli-Mpungu et al (2020)	HIV	In-person group meetings	Group support psychotherapy	38.5	1%	12.0	1140	**0.51**	0.15, 1.71
Oranta et al (2010)	CVD	Individual meetings with mixed delivery	Interpersonal counseling	59.6	2%	18.0	103	**2.04**	0.36, 11.67
Orth-Gomér et al (2009)	CVD	In-person group meetings	Self-monitoring, cognitive restructuring, coping, stress reduction, improving social support	61.5	14%	108.0	237	**3.03**	1.27, 7.23
Powell et al (2010)	CVD	In-person group meetings	Self-monitoring, environmental restructuring, support from family and friends, cognitive restructuring, and relaxation	63.6	24%	48.0	902	**1.15**	0.83, 1.60
Pristipino (2019)	HIV	In-person individual & group meetings	Ontopsychological short-term psychotherapy	55.0	3%	60.0	100	**2.41**	0.21, 27.46
Ries et al (1995)	COPD	In-person group meetings	Education, psychosocial support, exercise training	62.6	39%	72.0	119	**1.54**	0.73, 3.25
Rodin et al (2018)	Cancer	In-person individual meetings	Supportive-expressive sessions for medical, social, and spiritual considerations (CALM)	59.1	15%	16.5	305	**0.66**	0.40, 1.06
Salem (2017)	Cancer	Home-based family support	Family-oriented support	10.7	3%	12.0	64	**4.69**	0.22, 101.4
Sebregts et al (2005)	CVD	Combination of in-person formats	Patient and partner, stress management and education	NR	1%	11.0	171	**0.37**	0.01, 9.11
Simpson (2008)	Cancer	In-person group meetings	Survivor support group, relaxation, stress management	49.5	2%	12.0	89	**0.20**	0.01, 4.39
Sinclair et al (2005)	CVD	Individual meetings with mixed delivery	Encouragement, support, advice, stress management, resuming activities, and socialization	75.0	9%	3.3	324	**1.09**	0.51, 2.35

Sjobom et al (2017)	All outpatient	Telephone meetings	Motivational conversations, self-care support, patient education, and social/medical service coordination	NR	NR	12.0	390	**1.00**	0.22, 4.52
Smeulders et al (2010)	CVD	In-person group meetings	Medical, social and emotional self-management skills	66.7	10%	12.0	317	**0.94**	0.44, 2.03
Smith et al (2011)	T2D	In-person group meetings	Peer support	64.6	4%	24.0	395	**0.64**	0.22, 1.83
Steel et al (2007)	Cancer	Individual meetings with mixed delivery	Supportive-expressive therapy, CBT, education, & pharmacological intervention	67.0	18%	9.0	28	**1.49**	0.78, 2.85
Stern et al (1983)	CVD	In-person group meetings	Group counseling	54.0	2%	15.0	64	**3.74**	0.15, 95.26
Stromberg et al (2003)	CVD	Individual at home visits	Education, social support to patient and family	77.5	26%	12.0	106	**3.78**	1.43, 9.97
Thompson (1991)	CVD	In-person small group meetings	Supportive-educative counseling	NR	5%	6.0	60	**2.07**	0.18, 24.15
Vahedian-Azimi et al (2016)	CVD	In-person individual meetings	Family-Centered Empowerment Model	61.4	4%	30.0	70	**2.21**	0.19, 25.64
van der Meulen et al (2013)	CVD	In-person individual meetings	Counseling to manage physical, psychological, and social consequences	60.4	7%	12.0	205	**1.01**	0.34, 2.99
van der Spek et al (2018)	Cancer	In-person group meetings	Supportive group therapy	57.2	0%	6.0	170	**1.00**	0.02, 50.40
Wade et al (2019)	All outpatient	In-person individual meetings	Stress support, relaxation	58.6	7%	6.0	786	**0.90**	0.53, 1.55
Xavier et al (2016)	CVD	Combination of formats	Tailored discussions, family support focused on adherence	56.4	5%	12.0	805	**0.82**	0.43, 1.55

Abbreviations: *N* = Number of participants; *OR*, Odds Ratio; 95% CI, 95% Confidence Interval; NR, Not Reported; CBT, Cognitive behavioral therapy; CVD, Cardiovascular Disease; COPD, Chronic Obstructive Pulmonary Disease; CRD, Chronic Respiratory Disease; T1D, Type 1 Diabetes Mellitus; T2D, Type 2 Diabetes Mellitus; HIV, Human Immunodeficiency Viruses
