# Supplementary material for: Effects of psychosocial support interventions on survival in inpatient and outpatient healthcare settings: A meta-analysis of 106 randomized controlled trials
Source: PLoS Med. 2021 May 18;18(5):e1003595. doi: 10.1371/journal.pmed.1003595 (PMC8130925; doi:10.1371/journal.pmed.1003595)
Supplement: S3 Table — HR, hazard ratio. (PDF) [file pmed.1003595.s016.pdf]

S3 Table. Characteristics of 22 psychosocial intervention studies reporting hazard ratios of medical patient survival .

Source	Preexisting Condition	Intervention Format	Intervention Components	Avg. Age at Intake	Percent Died	Study Length (Months)	*N*	*HR*	95% CI
Andersen et al (2008)	Stroke	In-person group meetings	Stress reduction, health behaviors, family support	50.8	24%	144	227	**1.96**	1.08, 3.57
Blumenthal et al (2006)	LT	Telephone meetings	Supportive coping skills training	50.0	11%	43.8	272	**1.00**	0.46, 2.17
Choi et al (2012)	Cancer	In-person individual meetings	Problem solving, communication, counseling, and support	59.6	53%	90	230	**1.07**	0.75, 1.53
Cowan et al (2001)	CVD	In-person individual meetings	Relaxation, emotional coping, health education	NR	8%	24	133	**2.63**	0.75, 9.21
Edelman (1999)	Cancer	In-person group meetings	Cognitive skills, coping/relaxation, social communication training	49.7	91.0	60	92	**1.00**	0.64, 1.57
Giese-Davis et al (2011)	Cancer	In-person group meetings	Supportive-expressive group therapy	53.5	9%	12	101	**1.13**	0.70, 1.84
Goodwin et al (2001)	Cancer	In-person group meetings	Supportive-expressive group therapy	50.2	32%	15	235	**1.23**	0.88, 1.72
Gulliksson et al (2011)	CVD	In-person group meetings	Education, self-monitoring, communication skills training, cognitive restructuring, spiritual development	61.5	13%	144	362	**1.39**	0.77, 2.50
Härter et al (2016)	All outpatients	Telephone meetings	Motivational coaching for adherence, coping	69.4	5%	24	6125	**1.77**	1.38. 2.27
Hossain et al (2020)	All outpatients	Telephone and individual at home meetings	Monitoring, emotional support, self-care, supporting families	32.4	8%	24	410	**1.07**	0.53, 2.17
Jaarsma et al (2008)	CVD	Individual at home visits	Basic support, intensive support for adherence	71.0	27%	18	683	**1.18**	0.87, 1.59
Ilnyckyj et al (1994)	Cancer	In-person group meetings	Supportive educational group	50.0	64%	132	158	**1.00**	0.40, 2.48
Kissane et al (2004)	Cancer	In-person group meetings	Supportive group therapy, relaxation	46.3	NR	29	303	**0.73**	0.41, 1.30
Kissane et al (2007)	Cancer	In-person group meetings	Improve relationships, coping skills	51.7	52%	24	227	**0.95**	0.71, 1.27
Küchler et al (2007)	Cancer	In-person individual meetings	Psychotherapeutic support	50.0	58%	120	271	**1.49**	1.13, 1.96
Lin et al (2017)	CVD	Combination of in-person formats	Motivational interviewing, psychoeducation, coping, reminders	75.5	8%	18	288	**2.63**	1.09, 6.33

Orth-Gomér et al (2009)	CVD	In-person group meetings	Behavioral control, emotional coping, improve social support	61.5	14%	108	237	**3.23**	1.35, 7.69
Ross et al (2009)	Cancer	Individual at home meetings	Emotional support, information	68.5	59%	60	249	**0.93**	0.67, 1.31
Saab et al (2009)	CVD	Combination of in-person formats	Individual and group therapy focused on depression and perceived social support	60.6	NR	48	1503	**1.30**	0.94, 1.79
Spiegel et al (2007)	Cancer	In-person group meetings	Supportive-expressive group therapy	53.2	86%	168	125	**0.93**	0.62, 1.40
Spiegel et al (1989)	Cancer	In-person group meetings	Supportive-expressive group therapy	54.7	96%	120	86	**2.34**	1.52, 3.59
Stagl et al (2015)	Cancer	In-person group meetings	CBT, coping, optimize social support, relaxation	50.3	13%	180	240	**4.76**	1.10, 20.54

Abbreviations: *N* = Number of participants; *HR*, Hazards Ratio; NR, Not Reported; CBT, Cognitive behavioral therapy; CVD, Cardiovascular Disease; COPD, Chronic Obstructive Pulmonary Disease; LT, Lung Transplant
