# Supplementary figures and images for: Effects of psychosocial support interventions on survival in inpatient and outpatient healthcare settings: A meta-analysis of 106 randomized controlled trials
Source: PLoS Med. 2021 May 18;18(5):e1003595. doi: 10.1371/journal.pmed.1003595 (PMC8130925; doi:10.1371/journal.pmed.1003595)

**S1 Figure. Forest plot of 56 social/emotional support focused RCTs reporting odds ratios.**

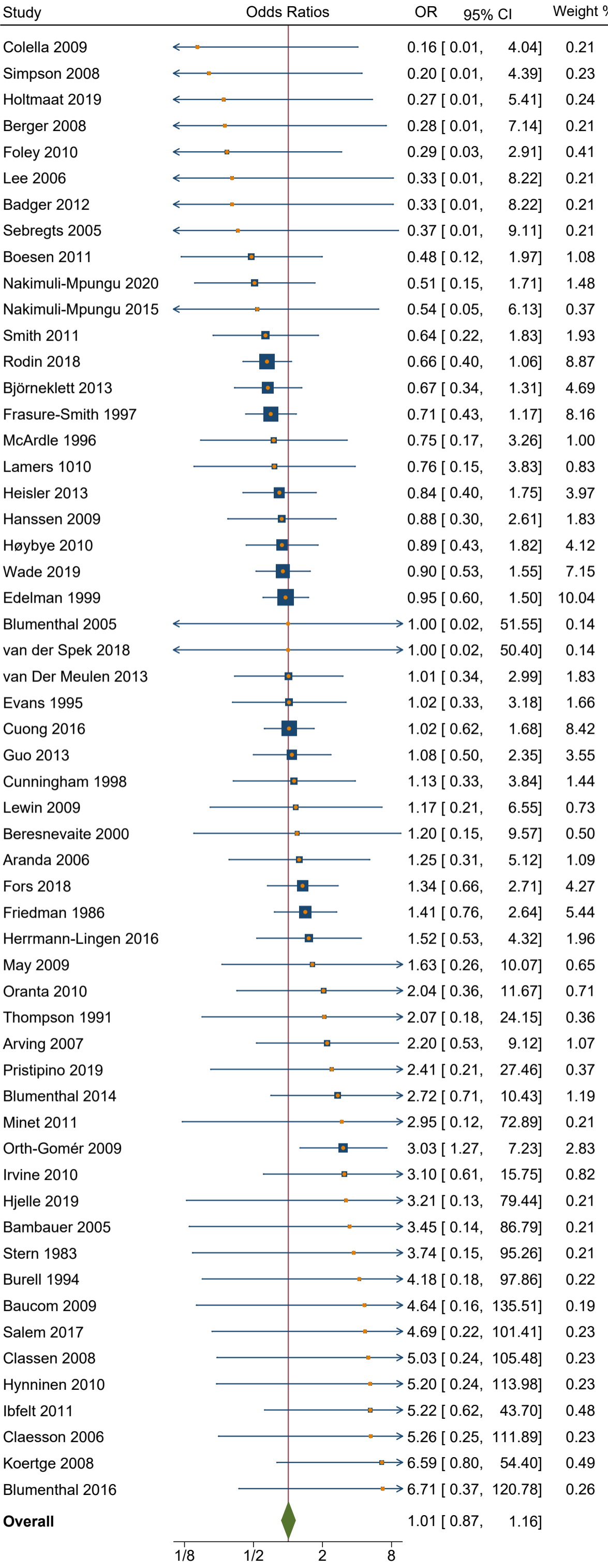

Supplement: S1 Fig — OR, odds ratio; RCT, randomized controlled trial. (PDF) [file pmed.1003595.s017.pdf]

**S2 Figure. Forest plot of 31 behavioral support RCTs reporting odds ratios.**

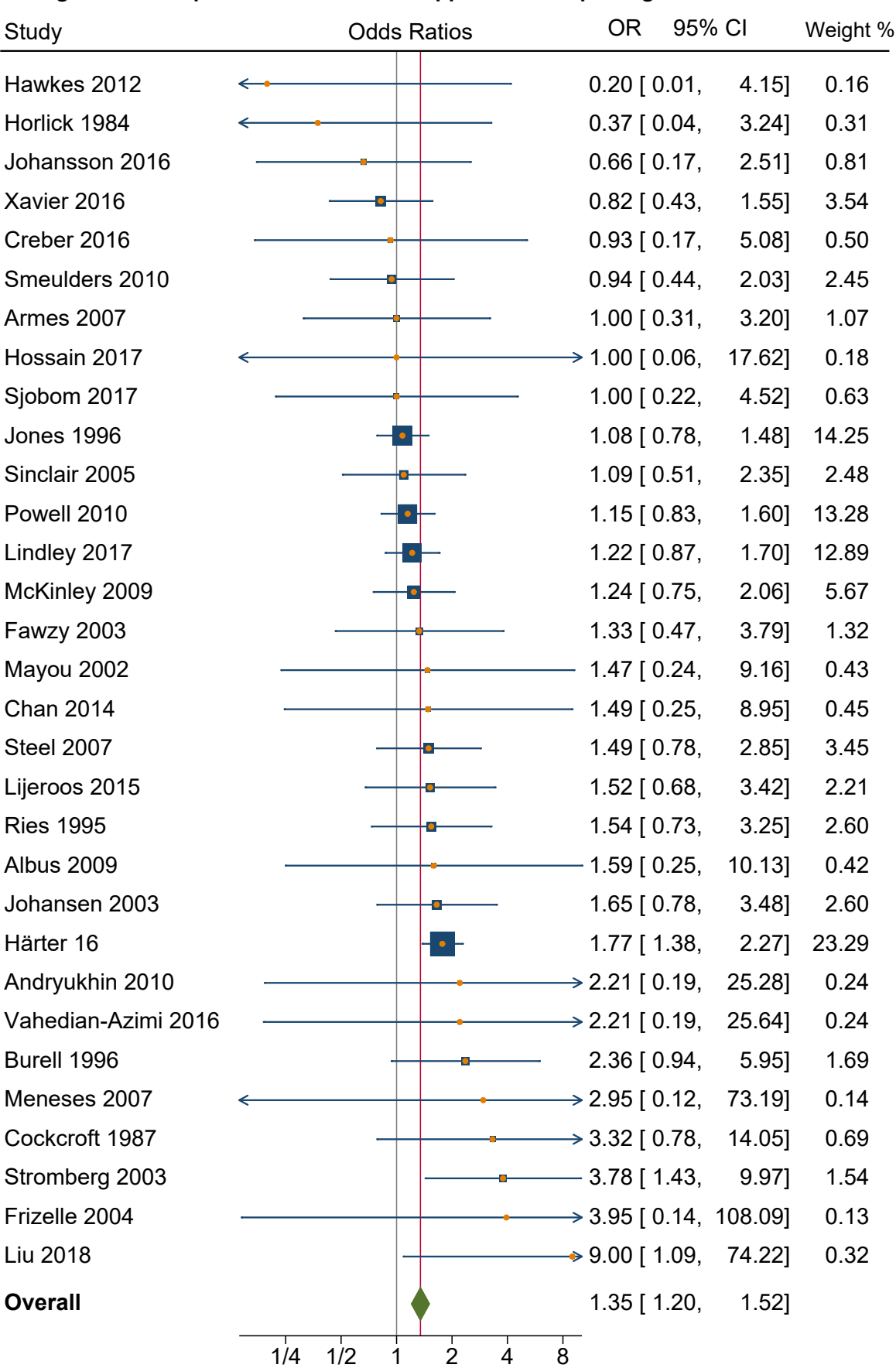

Supplement: S2 Fig — OR, odds ratio; RCT, randomized controlled trial. (PDF) [file pmed.1003595.s018.pdf]

**S3 Figure. Forest plot of 22 RCTs reporting hazard ratios.**

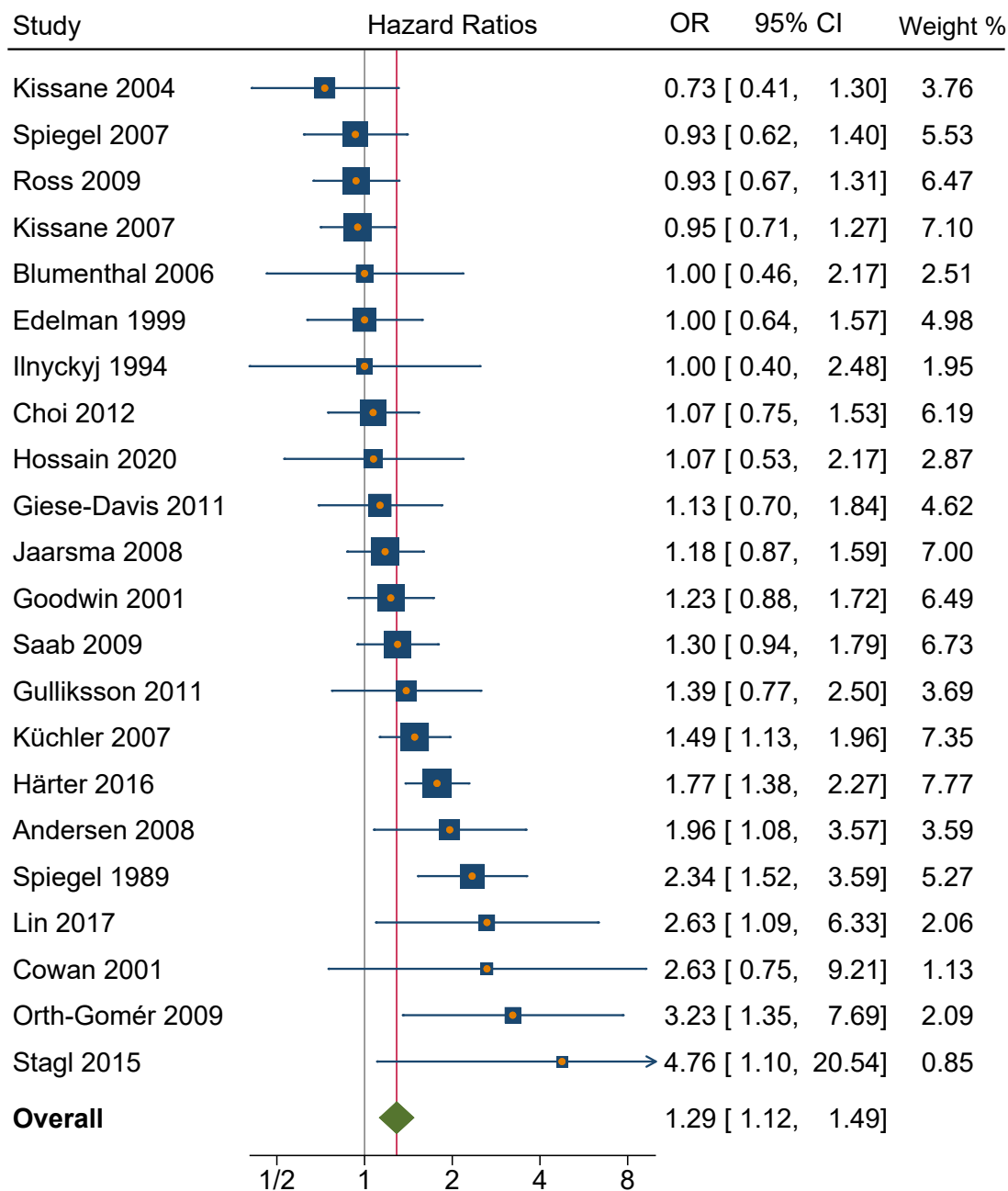

Supplement: S3 Fig — HR, hazard ratio; RCT, randomized controlled trial. (PDF) [file pmed.1003595.s019.pdf]

S5 Figure. Contour-enhanced funnel plot of 89 RCTs, odds ratio data.

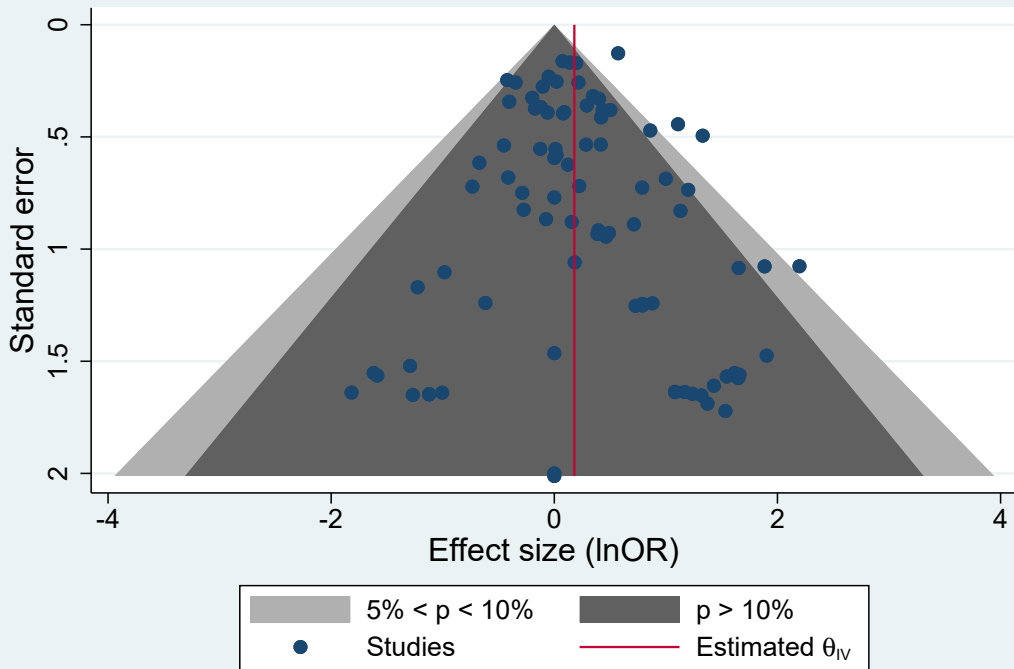

Supplement: S5 Fig — OR, odds ratio; RCT, randomized controlled trial. (PDF) [file pmed.1003595.s021.pdf]

S6 Figure. Contour-enhanced funnel plot of 22 RCTs, hazard ratio data.

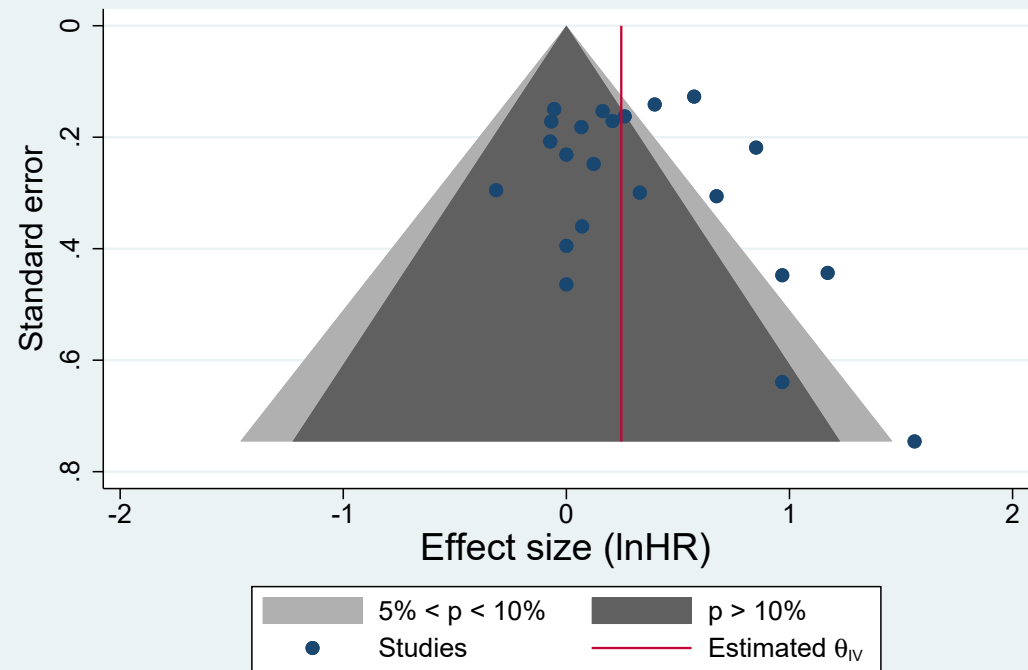

Supplement: S6 Fig — HR, hazard ratio; RCT, randomized controlled trial. (PDF) [file pmed.1003595.s022.pdf]
