# Supplementary material for: Effects of psychosocial support interventions on survival in inpatient and outpatient healthcare settings: A meta-analysis of 106 randomized controlled trials
Source: PLoS Med. 2021 May 18;18(5):e1003595. doi: 10.1371/journal.pmed.1003595 (PMC8130925; doi:10.1371/journal.pmed.1003595)
Supplement: Attachment — Submitted filename: Response to requested revisions PLOS Medicine March 2021.pdf [file pmed.1003595.s024.pdf]

Responses to Requested Revisions PMEDICINE-D-20-03297R2

1. Please revise the title to indicate that the meta-analysis was restricted to studies where recruitment occurred in health care settings, e.g. "Psychosocial support interventions delivered in inpatient and outpatient health care settings: a meta-analysis of 106 randomized controlled trials" Please ensure that the Abstract similarly emphasizes this distinction.

We have revised the abstract and title to indicate inpatient and outpatient health care settings: "Effects of psychosocial support interventions on survival in inpatient and outpatient health care settings: a meta-analysis of 106 randomized controlled trials."

In the abstract, we clarified “support interventions in inpatient and outpatient health care settings” (line 25)

2.Data availability statement: Thank you for making the study data available. Please provide a complete link to the dataset here (for example: https://osf.io/3qydb/)

We now include the complete link https://osf.io/3qydb/ (line 207)

3.Abstract: Conclusions: Line 57: The link to the dataset registration can be removed from the abstract.

We removed the sentence about dataset registration.

4.Throughout: Please remove spaces from within brackets of in-text citations ([1,2] instead of [1, 2] for example)

We removed all spaces within brackets of in-text citations.

5.Introduction: Line 140: Please revise to “sought to evaluate”

We revised the text to “sought to evaluate” (line 140)

6.Methods: Line 152: Please include a reference to the supporting information file containing the PRISMA checklist (S1_Checklist)

We inserted (S1 PRISMA Checklist) on line 153.

Methods: Line 170/Table 1: Please include a very brief rationale for excluding palliative care interventions, in the most appropriate point in the text (perhaps drawing on the most relevant points from the updated cover letter).

“We similarly excluded hospice or palliative care interventions which deserved separate review because of their focus on improving quality of life, not necessarily length of life, which is the observed outcome of this meta-analysis specific to curative and rehabilitative care.” (lines

176-179)

7.Methods: Data analysis section: The use of the first person here is fine; however, if possible we suggest considering a reduction in the use of “We…” as this seems to start the beginning of most sentences.

We replaced three instances of “we…” in the data analysis section. (lines 187-192)

8.Table 1: Please consider if this information needs to be displayed in a table format, or can be presented as subsections within the text of the Methods section.

We copied much of the text from Table 1 to the text of the Methods section. We moved details previously reported in Table 1 to S1 Table.

9.Figure 1: Please provide a more descriptive title/legend for the flow diagram.

Fig 1. PRISMA flow diagram of study selection process. (line 223)

10. Figure 2: Please provide a more descriptive title/legend for the diagram illustrating risk of bias.

Fig 2. Risk of bias graph of characteristics across 106 studies. (line 332)

11. Results: Description of included studies: Please provide the numerators/denominators (or at least the number, as the total number of studies, 106, is given earlier in the paragraph) for any percentages reported where the numbers aren’t displayed in a table.

We now provide numbers as well as percentages. (lines 213-235)

12. Results: Main analyses: Please report $p<0.001$ (rather than $p=0.0001$ at line 248, for example) unless there is a reason to report them to this number of digits.

We changed all values to $p < 0.001$

13. Results: Line 344-345 and Line 348-349: Please provide the p value in addition to the 95% CIs for the publication bias analysis for the 4 and 8 imputed studies from the HR and OR data.

We report the p values on lines 352 and 355

14. Page 24: Please remove the Competing Interests and Funding sections from the main text, and ensure that all information is accurately entered in the “Competing Interests” and “Financial Disclosures” section of the manuscript submission system.

We moved those two sections from the text and reported them in the submission system.

15. Reference list: Please use the "Vancouver" style for reference formatting, and see our website for other reference guidelines https://journals.plos.org/plosmedicine/s/submission-guidelines#loc-references

We have edited the references to “Vancouver” style formatting and followed the PLOS Medicine reference guidelines.

16. S1 Appendix: It would be helpful to provide the search criteria, analysis plan, and PRISMA checklist as separate files.

We now provide separate supplemental files for all components requested.

17. S2 Appendix: Please provide legends in addition to titles for all figures and tables, including those in Supporting Information files. Again, it would be helpful for each table/figure to be a separate file, to make it easier to refer the reader to the correct figure within the text.

We now provide separate supplemental files for all components requested.

Other changes to the text not reported above:

(A)

In the conclusion of the abstract, the previous version did not mention the main finding of *HR* data (only mentioning two moderation effects). We therefore clarified the interpretation as follows:

“HR data indicated that psychosocial interventions predominantly focused on social or emotional outcomes improved survival but yielded similar effects to health information/classes and were less effective among patients with apparently greater disease severity.” (lines 54-56)

(B)

In the Author Summary, a former statement inaccurately indicated “29% longer survival time” when the

HR data instead indicate likelihood of survival across time. So we changed the text to:

“and had 29% increased likelihood of longer survival, but results varied widely across studies.” (lines 73-75)

(C)

In the Data Analysis section, we removed the following sentence because although we routinely contacted authors when needed, we could not verify that the task was performed for all studies: "We contacted authors when relevant articles did not clearly compare survival data across groups."

(D)

In the first sentence of the Discussion section, we clarified "medical patients receiving curative or rehabilitative care." (lines 361-362)

(E)

In the Discussion section, we add a clarification that "The *HR* data predominantly represent interventions focused on social/emotional outcomes (18 of 22 studies)." (lines 382-383)

(F)

In the Discussion section, we added a phrase that research by other scholars has suggested a "focus on strengthening naturally-occurring relationships" (lines 463-464)
