# Supplementary material for: Effects of psychosocial support interventions on survival in inpatient and outpatient healthcare settings: A meta-analysis of 106 randomized controlled trials
Source: PLoS Med. 2021 May 18;18(5):e1003595. doi: 10.1371/journal.pmed.1003595 (PMC8130925; doi:10.1371/journal.pmed.1003595)
Supplement: Attachment — Submitted filename: Responses to Editor PLOS Medicine revisions Jan 2021.docx [file pmed.1003595.s023.docx]

| **Editor’s Comments** | **Authors’ Response** |
| --- | --- |
| 1.Prospective analysis plan: Did your study have a prospective protocol or analysis plan? If so, please provide the analysis plan as a supporting information file. Please state whether analyses were pre-planned (either way) early in the Methods section. | Our study protocol is reported in S1 Appendix. The prospective components were the literature search, inclusion/exclusion criteria, and methods for data abstraction. We pre-planned the omnibus analyses and publication bias estimates, but we did not pre-specify which variables to include in meta-regressions: we clustered variables logically by study and intervention characteristics.  The analyses reported in this version of the manuscript are the same as those reported in the previous manuscript, with two exceptions: (a) Based on the comments of the 1^st^ (statistical) reviewer, we shifted several variables previously reported in subgroup and sensitivity analyses to a third meta-regression specific to participant characteristics. (b) We report a subgroup analysis based on the first comment of the 2^nd^ reviewer, who proposed that health behavior change is the most likely causal influence.  In the revised manuscript text, we inserted the following:  Line 152  "see S1 Appendix for all study design information"  Lines 191-201  “Our data analysis plan (S1 Appendix) was to (a) report descriptive statistics of study characteristics, (b) calculate random effects weighted omnibus *HR* and *OR* values and also indicators of between-study heterogeneity (*Q* and *I^2^*), (c) conduct subgroup analysis across intervention type (behavior focused versus social/emotional focused), (d) report meta-regressions separately for study, intervention, and patient characteristics, and (e) estimate the likelihood of publication bias. We did not pre-specify which variables to include in the meta-regressions but clustered them according to study, intervention, and participant characteristics. We reported a subgroup analysis contrasting behavioral support with social/emotional support as a result of reviewer feedback, not as a pre-specified analysis. We prospectively planned to evaluate the likelihood of publication bias estimates using funnel plots, the trim and fill method, and Egger’s and Peters’ regression tests.” |
| 2.Data availability statement: Thank you for agreeing to make your data available. If the data are freely or publicly available, note this and state the location of the data: within the paper, in Supporting Information files, or in a public repository (include the DOI or accession number). If the data are owned by a third party but freely available upon request, please note this and state the owner of the data set and contact information for data requests (web or email address). Note that a study author cannot be the contact person for the data. | Data are freely/publicly available at <https://osf.io/3qydb>  Line 202  “… with data available at osf.io/3qydb.” |
| 3.Abstract: Background: The final sentence should clearly state the study question. | Abstract, Lines 19-22  “This meta-analysis addresses the questions of how effective are psychosocial support interventions in improving patient survival and which potential moderating features are associated with greater effectiveness.” |
| 4.Abstract: Methods and Findings: Please briefly summarize the patient demographics of those included studies. | Abstract, Lines 32-33  “Patient average age was 57.2 years, with 52% females and 48% males and with 42% having cardiovascular disease, 33% cancer, and 22% other conditions.” |
| 5.Abstract: Methods and Findings: Please provide p values for these results, also please provide more description of the results (for example, what are the OR/HR representing here: “Across 112 RCTs reporting data for discrete time periods, the average was OR = 1.15 (95% CI = 1.03 to 1.29; I2 = 23.6, I2 CI 4 to 40). Across 29 RCTs reporting survival time, the average was HR = 1.32 (95% CI = 1.15 to 1.51; I2 = 53.6, I2 CI 29 to70).”) | Abstract, Lines 33-40  “Across 87 RCTs reporting data for discrete time periods, the average was *OR* = 1.20 (95% CI = 1.09 to 1.31, *p* < 0.001), indicating a 20% increased likelihood of survival among psychosocial support recipients compared to those receiving standard care… Across 22 RCTs reporting survival time, the average was *HR* = 1.29 (95% CI = 1.12 to 1.49, *p* < 0.001), indicating a 29% increased probability of survival over time among social support recipients compared to those receiving standard care.” |
| 6.Abstract: Methods and Findings: In the last sentence of the Abstract Methods and Findings section, please describe the main limitation(s) of the study's methodology. | Abstract, Lines 47-48  “The main limitation of the data is that interventions very rarely blinded personnel and participants to study arm, such that expectations for improvement were not controlled.” |
| 7.Abstract: Conclusions: Please address the study implications without overreaching what can be concluded from the data; the phrase "In this study, we observed ..." may be useful. It is not clear what is meant by “interventions minimally improved patient survival…” in the first sentence- please clarify this to either state that the interventions did improve, or did not improve survival. | Abstract, Lines 50-56  “In this meta-analysis we observed that psychosocial behavioral support interventions (promoting patient motivation/coping to engage in health behaviors) improved patient survival, but interventions that primarily focused on patients’ social or emotional outcomes did not prolong life. We further observed that interventions tended to be less effective among patients with apparently greater disease severity.” |
| Please interpret the study based on the results presented in the abstract, emphasizing what is new without overstating your conclusions. “Psychosocial support programmes can be improved by better meeting patient needs.” this sentence does not seem to be supported by the study’s data or objectives. | We now interpret the study based on the results. We deleted the sentence quoted (which no longer applies). |
| 9.Author Summary: At this stage, we ask that you include a short, non-technical Author Summary of your research to make findings accessible to a wide audience that includes both scientists and non-scientists. The Author Summary should immediately follow the Abstract in your revised manuscript. This text is subject to editorial change and should be distinct from the scientific abstract. Please see our author guidelines for more information: <https://journals.plos.org/plosmedicine/s/revising-your-manuscript#loc-author-summary> | Lines 60-90  We have provided an Author Summary |
| 10.References throughout: For in-text citations, please place the reference number in brackets before the punctuation, like this [1]. | All references now appear in brackets. |
| 11.Introduction: Line 77-78: “Thus, in conducting the largest meta analytic review to date…” Please qualify this with “to the best of our knowledge” or similar. | Introduction, Lines 137-139  “Thus to evaluate differences across contexts, we have conducted what to our knowledge is the largest meta-analytic review to date, including three times the number of studies of any prior meta-analysis that we could locate on the topic.” |
| 12.Methods: Line 88: Please revise this to “This study is reported as per the Preferred Reporting Items for Systematic Reviews and Meta-Analyses (PRISMA) guideline.” Please provide the completed PRISMA checklist as a supporting information file. When completing the checklist, please use section and paragraph numbers, rather than page numbers. | Methods, Lines 151-152  “This study is reported as per the Preferred Reporting Items for Systematic Reviews and Meta-Analyses (PRISMA guidelines [44]; see S1 Appendix for all study design information).”  We reported section and paragraph numbers in the checklist. |
| 13.Methods Line 91-92: Please update your search to the present time. | Methods, Line 155  “…searches of studies from January 1980 to October 2020…” |
| 14.Results: Lines 146-148: Please provide the p values associated with “Across 112 estimates using fixed time periods, the average was OR = 1.15 (95% CI = 1.03 to 1.29), indicating a small increased likelihood of survival for intervention participants compared to controls. | We report *p*-values throughout the manuscript. |
| 15.Results: Lines 156-157: Please provide the OR/HR data with 95% CIs and p values for the findings excluding terminally ill patients or deaths shortly after baseline, between inpatients and outpatients, by health condition, and attrition vs. mortality, or treatment as usual with our without additional materials. Please refer to the table where these are presented. | Results  We report 95% CIs and *p*-values.  Based on the 1^st^ (statistical) reviewer’s recommendations, we report meta-regressions, with the results in Tables 3, 4, and 5. The variables “excluding terminally ill patients or deaths shortly after baseline” had previously been reported as subgroup analyses, but we could not include those variables in a meta-regression because they overlap with the total risk of bias estimate that includes the variable “intent-to-treat analyses.” We made that decision before conducting the revised meta-regressions (we have not analyzed those variables in the updated dataset). |
| 16.Results: Lines 167-168: Please also present the HR and 95% CI breakdown for this result, that intervention length was not associated with survival time. | Results, Tables 3-5  Intervention length and other variables previously reported as subgroup analyses are now reported in the meta-regressions, as per the feedback of the 1^st^ (statistical) reviewer. |
| 17.Results: Lines 171-174: Please provide the p values for the sensitivity analyses of the high quality studies. | Results, Tables 3-5  Study quality (risk of bias) and other analyses are now reported in the meta-regressions, as per the feedback of the 1^st^ (statistical) reviewer. We report all associated *p*-values. |
| 18.Results: Lines 177-181: Please provide p values for psychosocial functioning results. | Results  We report *p*-values.  Improvement in psychosocial functioning and other variables are now reported in the meta-regressions, as per the feedback of the 1^st^ (statistical) reviewer. (In our previous manuscript we had used the term “intervention effectiveness” to describe that variable, but this version uses the clearer terms “patient improvement on psychosocial variable(s)” or “psychosocial improvement”). |
| 19.Discussion: Line 316: The reference to the “current pandemic” should be removed. | Discussion  All references to the “current pandemic” have been removed. |
| 20.Figure 4: Please define the abbreviations for OR, HR, CVD in the figure legend. | The legend for Figure 3 (formerly Figure 4) defines the abbreviations. |
| 21.Competing interests: Can you please clarify the existing competing interests of the authors? The sentence “no financial relationships with any organisations that might have an interest in the submitted work in the previous three years” is not clear. | Competing interests, Lines 479-482  “[We] declare: (a) no support from any organisation for the reported work, (b) no relationships with any organisations that might have a financial interest in the reported work, and (c) no other relationships or activities that could appear to have influenced the reported work.” |
| 22.Supporting information: Please provide titles and legends for each individual table and figure in the Supporting Information. | We provide titles and legends for tables and figures in the Supporting Information (S1 Appendix and S2 Appendix). |

| **Academic Editor’s comments** | **Authors’ responses** |
| --- | --- |
| 1. The Introduction seems to conflate several different constructs (eg., social isolation, loneliness, and social support). And those are different from other constructs in the literature. eg. Social integration and social participation. As a result, it is unclear to me exactly what kinds of interventions are the focus of this study. The title and Methods refer to "psychosocial support" interventions, but when I look at the list of included studies, I see several CBT interventions (eg., Choi et al ref #63)-- but I would not characterize CBT as a "social support intervention". When I get to the Limitations and find out (line 280) that only 8/140 RCTs focused on naturally-occurring relationships, then I am even less certain as to what kinds of interventions were studied. ENRICHD, for example, was focused on getting study participants to strengthen their existing relationships, and is generally regarded as a prototypical "social support intervention". Does this mean that 132/140 RCTs were more of the "prescribe a friend" type? Lines 134-139 indicate that most interventions were more of the "clinician-delivered support" type. | Thank you for pointing out the need for greater clarity. There is indeed a variety of types of interventions, as social support theory includes emotional, informational, tangible, and belonging support. We recognized that some interventions provide education to patients (informational support) but little else. Therefore, we focused on those that provided emotional support or tangible support, which typically came in the form of behavioral coping with illness. This is now clarified on Lines    Given the classic studies by Spiegel and colleagues of cancer social support groups, we knew we could not restrict analyses to support for naturally-occurring relationships. To address this issue, we pre-planned and coded for whether studies involved family/partner – or group peer support, with that analysis reported in Table 4.    As for the inclusion of CBT interventions, we agree that these are not necessarily social support. Some do focus on maladaptive thinking that interferes with social interactions. Indeed, in a meta-analysis of interventions for reducing loneliness (Masi et al, 2011), CBT was one of the most effective. However, we recognize that not all CBT focused on interpersonal or emotional coping – and that one-on-one psychotherapy is a distinct type of support deserving *separate* review.  Based on the Academic Editor’s observations about (a) the problematic nature of CBT interventions, (b) method of delivery (existing vs. new relationships), and (c) the need to clarify inclusion/exclusion criteria (also mentioned by other reviewers), we made multiple revisions:   - We revised the wording of the introduction section by removing concepts reported by other scholars that were not directly relevant to this meta-analysis. - We more clearly specify the inclusion/exclusion criteria and report details in Table 1. - We removed studies solely providing CBT to individual patients; we similarly excluded all one-on-one psychotherapy studies based on the second comment of the third reviewer. We included CBT when it was one component of a larger intervention that had other components that clearly met inclusion criteria (e.g., family meetings). We retained *group* psychotherapy interventions, since group support facilitated by a therapist is similar to group support facilitated by medical staff or peer survivors. - We report the following in the Results section, Lines 223-225 “Interventions were conducted by nurses or medical staff (36%), social workers or mental health professionals (30%), peers with the same medical condition (4%), or combinations of those groups (22%), with 8% unspecified. Across all interventions, 63% intended to foster patient social relationships with previously unknown individuals; about 19% provided support only from professional staff; and 33% invited family members—only 9% focused on family-patient relationships.” |
| 2. The search needs to be updated. My understanding is that PLOS generally requests for evidence searches to be out of date by no more than 6 months. Should this manuscript make it through peer review, by the time it is published, more than 3 years will have elapsed since the evidence search was conducted. | Abstract, Line 26  Method, Line 156  Table 1, Line 174  We updated the literature search through the end of October, 2020. |
| 3. Line 98: The authors restricted analysis to studies of "medical patients". Perhaps they could be more specific about what this term means. Are they referring to inpatients? Outpatients? Or did the included studies need to include study participants with a defined medical diagnosis and/or acute medical illness? (Line 104 ["mental illness as the sole health condition"] seems to imply that included studies were focused on some health conditions.) Any study conducted with a participant sample recruited in a health care setting? If so, does this mean they excluded group interventions occurring outside of health care settings (eg., Alcoholics Anonymous)? I see "self-help group" in the search strategy but not "AA", "Alcoholics Anonymous", or "12 step" (and those terms would normally be included in a systematic review of AA-style interventions, cf. Kelly JF, Magill M, Stout RL. Addict Res Theory 2009;3(17):236-259). They seem to have included "home visits", so I would expect that AA-style interventions were also included--but the search strategy does not seem like it was designed to capture these.? | Table 1  “We included studies of patients with a health condition likely to result in death if untreated and who are recruited from health care settings (e.g., hospitals, clinics, inpatient/outpatient databases). We retained studies in which patients initially identified in health care settings received subsequent rehabilitation or home visits.”  We excluded studies initiated outside health care settings, including Alcoholics Anonymous and similar 12-step support groups. |
| 4. Lines 101-104: There are a few things that make the exclusions a bit "squishy". (a) The authors state that they are focused on "disease" but then exclude suicide, mental illness, dementia-- is the implication that mental illness is "not disease"? (b) If the authors excluded studies of mental illness as the "sole health condition", does that mean they included studies that were focused on depression among study participants with another medical condition? I see Berkman/ENRICHD in the list of included studies, so I assume this is true. Please clarify. (c) I am in agreement with R2 that the exclusion of palliative care interventions "not intended to prolong life" seems problematic and should be clarified or amended. | We are very grateful for the feedback to refine inclusion/exclusion criteria.  Method, Lines 167-171  "As we focused on the impact of psychosocial support among patients who received curative or rehabilitative care and who had the capacity to respond to treatment, we excluded (a) studies of mortality resulting from accident, suicide, or violence, (b) studies of dementia or mental illness as the sole health condition, and (c) studies of hospice/palliative care. Table 1 provides detailed criteria."  Table 1  “We excluded patients with solely mental health disorders (e.g., anxiety, dementia) because other than increased likelihood of suicide, accidents, and possibly violence, those conditions only indirectly contribute to death if untreated. We retained studies in which patients had mental health conditions in addition to physical conditions resulting in death if untreated. We excluded patients receiving hospice or palliative care since we believed those interventions deserved separate review.” |
| 5. Line 111: Does this mean that none of the studies reported data as (for example) standardized effect sizes, regression coefficients, etc.? I find this very, very hard to believe. In any case, if this is true, please indicate here in the text. | Lines 180-182  “Effect size data were hazard ratios (*HR*) and odds ratios (*OR*); when studies reported other values (e.g., regression coefficients, Cohen’s *d*), we transformed them to *OR* using multiple effect size calculators available online.” |
| 6. Line 115: What does "questionable effect size data" mean? Please be more specific here in the text. Examples (eg., in the Appendix) may help. | Line 186  We corrected that sentence to: “We contacted authors when relevant articles did not clearly compare survival data across groups.” |
| 7. Line 116 and elsewhere: The authors use the term "multivariate" but I believe they mean "multivariable". (cf. Peters TJ. Paediatr Perinat Epidemiol 2008;22(6):506). | Line 187  “We sought effect sizes from multivariable models but calculated odds ratios from survival frequency counts when statistical models were unreported.” |
| 8. Line 116: The authors included randomized controlled trials. Why would these studies need to "control for known confounds"? Are the authors referring to randomized studies in which covariate balance was not achieved through randomization? Please clarify here in the text. | We removed the phrase "that controlled for known confounds (e.g., health status, age)." |
| 9. Line 129 seems to suggest that studies were restricted to either medical inpatients or medical outpatients. (cf comment #3 above) | We included studies of inpatients and outpatients. Inclusion criteria reported in Table 1 now indicate:  “We included studies of patients with a health condition likely to result in death if untreated, and who were recruited from health care settings (e.g., hospitals, clinics, inpatient/outpatient databases). We retained studies in which patients initially identified in health care settings received subsequent rehabilitation or home visits.” |
| 10. Abstract, Line 147, Line 236, Figure 4, etc.: why are the findings (pooled OR=1.15, pooled HR=1.32) described as "minimally" improving patient survival (eg., "small increased likelihood of survival", "increased odds to a small degree")? I am _not_ suggesting that the authors revise this adjective (eg., "substantially"). I am simply requesting that the authors justify their use of this adjective. I see the comparisons to exercise, cardiac rehab, etc. But I do not find those examples too compelling. Aspirin reduces the risk of CVD events by 15% in relative terms (ie, meta-analysis OR=0.85), but I do not believe that clinicians would characterize that as a minimal improvement. Perhaps in the literature on social support interventions the bar is different? | We agree with this observation about mortality data, and we removed all problematic adjectives (“minimally” and “small”). |
| 11. Line 155: All of these subgroup analyses, and the meta-regression, should be described in the Methods section. | Method, Lines 192-202  “Our data analysis plan (S1 Appendix) was to (a) report descriptive statistics of study characteristics, (b) calculate random effects weighted omnibus HR and OR values and also indicators of between-study heterogeneity (Q and I2), (c) conduct subgroup analysis across intervention type (behavior focused versus social/emotional focused), (d) report meta-regressions separately for study, intervention, and patient characteristics, and (e) estimate the likelihood of publication bias. We did not pre-specify which variables to include in the meta-regressions but clustered them according to study, intervention, and participant characteristics. We reported a subgroup analysis contrasting behavioral support with social/emotional support as a result of reviewer feedback, not as a pre-specified analysis. We prospectively planned to evaluate the likelihood of publication bias estimates using funnel plots, the trim and fill method, and Egger’s and Peters’ regression tests.” |
| 12. Line 158 has a typo ("statistically significant differ") | We corrected the typo. |
| 13. Line 169: I see the N's for the low-quality studies. What were the N's for the high quality studies, and what were the pooled estimates?  I did not see a Risk of Bias assessment table. This would be particularly important to know which studies (OR vs HR data) were in the "high risk of bias" vs "low risk of bias" groups, because the higher quality studies did not reach statistical significance.  How was risk of bias assessed? This is only mentioned in passing on line 109. eg Did a study get 1 point each for blinding, exclusions, attrition (what threshold?), and then "low risk of bias" = 3 points? | We now report a Risk of Bias graph in Figure 2 – and a Risk of Bias summary table in S2 Appendix. We evaluated multiple indicators of bias according to the Cochrane risk-of-bias tool (RoB 2): random sequence generation, allocation concealment, blinding of participants and personnel, blinding of outcome assessment, incomplete participant data, selective reporting, groups balanced at baseline, intent-to-treat analysis, and groups had same conditions, apart from intervention.  In our original manuscript, we had reported a categorical variable, with our analyses only including studies at high risk of bias (contrasted with all others). Recognizing the limitations of collapsing all ratings into two groups, we now report analyses based on the sum of all sources of bias. For each indicator of bias (e.g., allocation concealment), the data were coded: 0 = low risk of bias, 1 = unclear risk of bias, and 2 = high risk of bias. Summed across indicators, the total ranged from 0 to 9, with an average of 3.9 (*SD* = 2.1). We included this variable in the three meta-regressions (Tables 3-5). We report the pooled estimate for low risk of bias studies in Figure 3. |
| 14. Line 175 and Figures 2 & 3: what do the authors mean by "statistically significant improvements on psychosocial variables"? Presumably variables like depression symptom severity, social role functioning, etc. were included. What kinds of variables were excluded? What do the authors mean by "inconsistent/ineffective" in Figure 2? (What if a study was "consistent" but "ineffective", or "inconsistent" but "effective"?) I see that these data are partially represented graphically in Figures 2 & 3, but it would be helpful to have an Appendix table that is analogous to Table 2 showing each study line by line (stratified by "inconsistent/ineffective" vs. "effective"), the "psychosocial variable" assessed (eg., 15-item Hopkins Symptom Checklist), and the efficacy estimate and standard error on the psychosocial variable. | The variable previously called “intervention effectiveness” (now called “psychosocial improvement” for clarity) was coded: 0 = studies not reporting relevant data or demonstrating no statistically significant improvements on psychosocial measures administered, 1 = statistically significant improvements on at least one psychosocial measure but not on others, and 2 = statistically significant improvements on all psychosocial measures administered. We evaluated all psychosocial measure(s) reported by study authors (none excluded). This variable no longer reached statistical significance in the meta-regression, so we presumed that it would not be helpful to report subgroup data in an Appendix. |
| 15. Line 184: The average age and percentage of female patients are not study-level characteristics and should not be included in the meta-regression model. | We now report a meta-regression specific to patient characteristics (Table 5). |
| 16. Line 284: "many studies did not measure preexisting levels of support"-- I did not see this anywhere in the Results, please add. | Our previous wording was based on anecdotal observation, not on coded data. We now clarify in the Limitations section, Lines 429-430  "Fourth, we did not evaluate pre-existing levels of patient psychosocial support” |

| **1^st^ reviewer (statistical)** | **Authors’ responses** |
| --- | --- |
| First - As I was reading along, I thought "why aren't they doing meta-regression?" Unless I missed something, this was first mentioned on p 8. It ought to be mentioned in methods on p. 4 or 5. (The authors say 'subsequent analysis' but that is very vague). | We now report conducting meta-regressions.  Abstract, Line 41  "Among those studies, meta-regressions identified three moderating variables..."  Method, Lines 196-198  “…report meta-regressions separately for study, intervention, and patient characteristics, and (e) estimate the likelihood of publication bias. We did not pre-specify which variables to include in the meta-regressions but clustered them according to study, intervention, and participant characteristics.” |
| Lines 50 and 52: It isn't clear what these OR and HR are, exactly. It seems like it is "support of any kind" vs. "no support" but this should be made clear. | Abstract, Line 34-41  “Across 87 RCTs reporting data for discrete time periods, the average was *OR* = 1.20 (95% CI = 1.09 to 1.31, *p* < 0.001), indicating a 20% increased likelihood of survival among psychosocial support recipients compared to those receiving standard care. Across 22 RCTs reporting survival time, the average was *HR* = 1.29 (95% CI = 1.12 to 1.49, *p* < 0.001), indicating a 29% increased probability of survival over time among social support recipients compared to those receiving standard care.”  Results, Lines 248 and 262  “…indicating a 20% increased likelihood of survival for intervention participants compared to controls.”  “…indicating a 29% increased likelihood of longer survival compared to controls.” |
| Line 101 What does the sentence starting "Differences were coded ..." mean? What differences? Coded how? | We replaced that very unclear sentence with several mentions of our plan to compare differences across study, intervention, and patient characteristics.  Lines 130, 166, 196 |
| Line 149 ff I don't think statistical significance of Isquared is particularly useful. Maybe delete the p values and CIs. | We no longer report the *p-*value or confidence interval for the *I^2^* statistic. |
| Subgroup analysis - I'm unclear on how subgroup analysis was used to conduct hypothesis tests and yield p values and so on. This is what meta-regression doe. But if you do an analysis only on one subgroup, how do you do a tst of it vs. another subgroup? This doesn't make the subgroup analysis wrong, but .... how did the authors get these results? | We shifted several variables previously reported in subgroup analyses to meta-regressions (Tables 3-5). Planned subgroup analyses evaluate categorical variables (random effects weighted *Q-*test based on analysis of variance [ANOVA]) when there is a theoretically compelling reason to compare groups and also when the number of studies is small (restricting the number of variables to include in a meta-regression). |
| Fig. 2 is way too small a font. Maybe spread it over several pages. | We now use adequate font size in figures. |
| Fig 3 I like that there is relatively little text here. But it would be good to add a vertical line at OR = 1 That would make it a lot clearer that these effects are in one direction. | The three Forest Plots reported in S2 Appendix now all have vertical lines at 1. |

| **2^nd^ Reviewer** | **Authors’ responses** |
| --- | --- |
| 1) In the introduction the authors aim to establish a link from patient loneliness and lack of social connections to the eventual meta-analysis of a wide range of psychosocial support interventions, including for example several-session telephone disease management programs. The authors need to better explain how and why such interventions would be able to reduce loneliness/increase social connectedness as the assumed mechanism of prolonging survival. It is hard to imagine how low-dosis interventions of this structure that have been included in the review could change an individuals' social relationships so enduringly that survival is significantly prolonged. Yet it seems reasonable to assume a different mechanism: in-person disease management or telephone support with home visits can improve health behavior (therapy adherence, smoking, diet, alcohol consumption etc.), which in turn improves survival. | Introduction  Thank you for bringing this to our attention. Indeed, patient loneliness and lack of social connection are broader issues that indicate potential lack of support among clinical patients. In the revised manuscript we focus more on the support in the clinical context.  We further describe a plausible link between social/emotional support and patient health:  Lines 120-126  “Abundant research evidence suggests that psychosocial distress co-occurs with physical disease, with bidirectional relationships that influence disease progression (e.g., appraisal, self-regulation ability). Research indicates that psychosocial functioning not only affects relevant social capital (e.g., access to health information, improved trust of healthcare) but can also reduce inflammation and improve systemic circulation. More specifically, even short-term emotional management interventions can influence inflammatory gene expression.”  Much of the social support literature does suggest that behavioral influence is a potential mechanism by which relationships influence health outcomes. We have now included additional details in the third paragraph of the revised manuscript. Lines  At a much broader level, this first recommendation from the second reviewer strongly influenced the overall revisions we undertook. The assertion that behavior interventions (e.g., supporting therapy adherence) provide the (sole) logical mechanism was compelling, although we questioned if that would be the case, given mounting support for the effects of social/emotional support. We shifted our manuscript revisions to address whether behavioral or social/emotional support is more effective in prolonging life. That plan proved problematic in that between-group heterogeneity was zero for *OR* studies – and only 4 of 22 *HR* studies focused on behavioral support. We contacted a (non-acquaintance) scholar who had published five articles on heterogeneity in meta-analysis and received this response: “You may not find anything with metareg if the estimates do not differ across covariates. Would it make sense to do a subgroup meta-analysis? You could say, if justified, *a priori* that you think XYZ groups make sense in the current context and thus you produce subgroup estimates. However, the estimates may not differ between subgroups. On the other hand, if theory suggests that XYZ grouping is in order, I think you can do this. Subgroup analysis is like meta-reg with only categorical variables. A justification based on theory should supersede an analysis based on the results/after looking at your data (post hoc). I hope this helps.” We affirm that we decided *prior to conducting the analyses*, based on strong theoretical rationale, to compare behavioral support with social/emotional support in subgroup analyses based on this recommendation received. |
| 2) Inclusion criteria: The authors state that interventions "not intended to prolong life" were excluded. This is a difficult statement because, at least for the majority of the included cancer studies, the study was not conducted with a primary intention of prolonging survival but to enhance quality of life. However, survival was co-assessed as one of many potential outcomes. This needs to be clearly stated. It is difficult to understand why similar studies in palliative care populations were excluded. For example, several early-palliative-care intervention trials reported survival data along a range of outcomes and they are not represented in this review. | We agree with the reviewer and clarified the wording of the inclusion/exclusion criteria: this meta-analysis is specific to curative and rehabilitative care. A difficulty that we encountered early in our project design was that although palliative care can co-occur with curative care, studies of palliative care did not always report the status of curative care. Moreover, palliative care focuses on improving quality of life, not necessarily length of life, even in studies in which survival data are reported. Although palliative care is appropriate at any stage of illness, studies often did not differentiate early- versus late-stage care (all patients receiving palliative care combined); and complications occur with late- and end-stage patients (e.g., ability and motivation to participate). Furthermore, the number of potential palliative care studies would have exceeded our resources to code data. We determined that palliative care deserved *separate* consideration prior to undertaking the meta-analysis. |
| Page 6, line 139: The average length of intervention sessions seems very long. Do the authors mean the total length of all sessions? | Results, Lines 229-230  We now clarify: “On average, each intervention session lasted 83 minutes (SD = 46.1; excluding one day-long intervention [98]).” These data were normally distributed: skewness = 0.62, kurtosis = 0.42.  Table 2 reports intervention length for OR and HR data separately. |
| Abstract/Results: It is difficult to understand, what exactly the OR of 1.15 means in terms of survival. I suggest adding a sentence explaining this in simple words. The addition of separate results for hazard ratio-studies increases the confusion. Please make both numbers more accessible by an explaining sentence. | Abstract, Lines 34-36  “Across 87 RCTs reporting data for discrete time periods, the average was OR = 1.20 (95% CI = 1.09 to 1.31, p < 0.001), indicating a 20% increased likelihood of survival among patients receiving psychosocial support compared to control groups receiving standard medical care.”  Lines 39-41  “Across 22 RCTs reporting survival time, the average was *HR* = 1.29 (95% CI = 1.12 to 1.49, *p* < 0.001), indicating a 29% increased probability of survival over time among intervention recipients compared to controls.” |
| Table 1/2: Please report descriptive data about time of survival or the time period studied for intervention and control condition for OR and HR studies. | Table 2 now contains descriptive data for OR and HR studies. |
| Table 1/2: The tables should also entail brief information about the content and type of the intervention in addition to the format. | S2 Appendix  S2 Table 1 and S2 Table 2 that report individual study characteristics now include information about the intervention type and content. |
| Table 1/2: The tables would also be easier to read if the confidence interval were included rather than or in addition to the standard error. | S2 Appendix  S2 Table 1 and S2 Table 2 that report individual study characteristics now include the 95% confidence interval rather than the standard error. |
| More information is needed on how the authors decided on whether a study was "effective" or "ineffective" regarding psychosocial outcomes. Most studies may have been ineffective with regard to some and effective with regard to other outcomes. | The variable previously called “intervention effectiveness” (now called “psychosocial improvement” for clarity) was coded: 0 = studies not reporting relevant data or demonstrating no statistically significant improvements on psychosocial measures, 1 = statistically significant improvements on at least one psychosocial measure but not on others, and 2 = statistically significant improvements on all psychosocial measures administered |
| Figures: Does effectiveness refer to psychosocial outcomes? Please include a note in the figure caption. | For clarity, we replaced the term “intervention effectiveness” with the term “Psychosocial improvement achieved,” and the footnote in Table 3 clarifies: “^1^Statistically significant improvement on psychosocial measures at the end of the intervention compared to controls.” |
| What size was the association between effectiveness in terms of psychosocial improvements and risk of bias? Did the authors control for multicollinearity of the two variables entered simultaneously to the regression models? | Patient psychological improvement correlated with total risk of bias *r* = 0.18. We now report that collinearity diagnostics indicated that multicollinearity did not characterize the three meta-regressions. |
| 3) A highly relevant result is that if high-quality studies were considered alone, no effect on survival was found. This is an important finding suggesting cautious interpretation. | We added a section specific to risk of bias: Lines 318-325  We included risk of bias in the three meta-regressions (Tables 3-5).  We also included cautions in the following locations:  Lines 56-57  “Risk of research bias remains a plausible threat to data interpretation.”  Lines 402-406  “the findings differed as a function of study risk of bias, with studies reporting more robust results also tending to have more indicators of research bias… the result provides a caution that qualifies the overall findings reported in the literature.”  Lines 453-456  “Given the concerns raised in this meta-analysis about study risk of bias adversely impacting the reported results, future research should specifically address that issue. Although blinding personnel and participants to the other study arm may be challenging, this gap needs to be addressed to advance the science beyond its current state.”  Lines 465-467  “this meta-analysis urges increased methodological rigor” |
| The conclusion to better meet patients' needs does not follow from the results. | We removed that phrase from the updated manuscript. |

| **3^rd^ Reviewer** | **Authors’ responses** |
| --- | --- |
| 1) In the first sentence, the consensus report calling for interventions for loneliness and social isolation in medical and health care settings is interesting, but the construct of social isolation and loneliness among persons dealing with chronic illnesses. While a small distinction, if the statement is true, then it might be helpful to change the reference to something more relevant. | We have revised the start of the manuscript.  Lines 95-101  “Decades ago researchers found that psychosocial support interventions (e.g., survivor groups, individual nurse support sessions) may improve not only patient quality of life but also patient survival. Subsequent evidence regarding patient survival has been mixed.  Adequate support among medical patients has been linked to better outcomes, while those that lack adequate support systems have poorer outcomes including greater hospitalization, mortality and medical costs – such that evaluations of supportive psychosocial interventions have been recommended in health care settings.” |
| 2) Some interventions contain content that focuses on enhancing support within the person's network using CBT (overcoming barriers to support). Other interventions are delivered by aj interventionist but would contain solely education and skill practice, but like most 1:1 treatments, a bond develops. Some group treatments are not support oriented, in that the group is more of an education delivery method, but the members obtain support after group or during group exercises in the room. Solely online interventions can contain supportive elements, via email exchanges/message boards, or even videotaped content offering normalization and narratives from other patients. Disease management support is categorized as support, but these are more educational interventions, unless I am missing something. Couples treatments may or may not include support, but may reduce conflict. It would have been very helpful to define social, emotional, and psychological support more clearly and discuss why disease management support is included. Disease management is assumedly to foster adherence to medical treatment. The supplemental materials define interventions but the above approaches would not be clearly included or excluded as the definition is very general.- | Based on this comment (and similar comments by other reviewers addressed above), we specified the inclusion/exclusion criteria (Table 1), including the criterion of real time interactions with humans. We also screened previously coded manuscripts to exclude studies solely providing one-on-one psychotherapy and disease management interventions, as mentioned previously. |
| 3) The conclusion that the interventions that resulted in psychological improvement achieved better outcomes in terms of survival than control interventions seems a little generic. Since the interventions may have provided support as well as coping skills (at least if my understanding of the interventions meeting criteria are correct), then the conclusion might state something more along the lines of "the intervention may not have included effective components." This leads me to the broader concern that the psychotherapeutic goals of each intervention might be better characterized in this study, as efficacy may be due to the components, and that would guide this study's implications much more clearly. | The former finding regarding psychosocial improvement no longer characterized the data.  Based on the comment regarding the probable importance of treatment *components* (and similar observations of other reviewers), we conducted the subgroup analysis of behavioral support vs. social/emotional support. |
| 4) It might have been interesting to discuss the baseline level of loneliness/low support as a moderator of effects for future studies that focus on survival. It may be that the interventions have a stronger impact among lonelier patients, and that may be why older patients fared better. | We did not evaluate baseline support, but we agree that this is an important consideration. The revised manuscript now states on Line 456-457:  “Other scholars have recommended that future research identify patient existing psychosocial supports and needs prior to intervention…” |
